# Dynamic cross-linked topological network reconciles the longstanding contradictory properties of polymers

**DOI:** 10.1126/sciadv.adt0825

**Published:** 2025-03-19

**Authors:** Zekai Wu, Chengzhen Chu, Yuhui Jin, Lei Yang, Bo Qian, Yuepeng Wang, Yihan Wang, Jiani Wu, Yujie Jia, Wenwen Zhang, Zhengwei You

**Affiliations:** State Key Laboratory of Advanced Fiber Materials, Institute of Functional Materials, College of Materials Science and Engineering, Donghua University, Research Base of Textile Materials for Flexible Electronics and Biomedical Applications (China Textile Engineering Society), Shanghai Engineering Research Center of Nano-Biomaterials and Regenerative Medicine, 2999 North Renmin Road, Shanghai 201620, China.

## Abstract

There is usually a trade-off between high-tensile properties and processability in polymers because the mechanisms of these properties are mutually exclusive. Here, we design a chemically coupled four-arm dynamic polymer cross-link site to overcome this challenge. By concurrently increasing cross-link sites and dynamic bond contents, this approach fabricates polymer networks with high cross-link density yet low processing temperature, challenging the conventional structure-property relationship where cross-linking inherently limits plasticity. Notably, the material demonstrates remarkable processability, evidenced by the ratio of *G***′**_max_ to *G***′**_min_ with a temperature differential (Δ*T*) of 120°C (which signifies the soft-to-hard transition capability). This ratio reaches 153.3, higher than all reported cross-linked polyurethanes. This work represents a molecular strategy that combines electronic effect and topology network design to modulate materials’ properties, and it will be useful for developing next-generation materials.

## Teaser

Increasing the dynamic cross-linking density simultaneously enhances both the tensile and processing properties of the materials.

## INTRODUCTION

Covalent network polymers, constituting a vital subclass of polymers with robust and enduring three-dimensional architectures, display distinctive characteristics comprising mechanical strength, thermal stability, and elastic recovery. These properties make them widely used in various industries (*1*). However, the persistent cross-linking in thermosets precludes their reprocessing, exacerbating the polymer industry’s carbon footprint as their disposal rates escalate.

In response, cross-linked polymers with dynamic covalent bonds, known as covalent adaptable networks (CANs), have emerged, wherein the covalent bonds within the network can spontaneously or, upon suitable stimuli (such as heat, catalysts, light, etc.), undergo reversible break and reformation of connections between polymer chains (*2*). Since the groundbreaking report of Wudl and co-workers (*3*) in 2002 on thermally repairable networks using the Diels-Alder reaction, researchers have explored various other reversible bonds [such as carbamate bonds (*4*, *5*) urea bonds (*6*, *7*), boron-oxygen bonds (*8*, *9*), and disulfide bonds (*10*, *11*)] for developing CANs. Although the introduction of dynamic covalent bonds enables the processing of cross-linked polymers, the ultimate goal of making mechanically superior polymers easier to process remains unattained. In general, enhancing material mechanical properties can be achieved by appropriately increasing cross-link density. However, as polymer physics pioneers James and Guth (*12*, *13*) noted, cross-linking restricts the plasticity of polymer chains, hindering effective reprocessing at high temperatures and thereby undermining the full potential of these CANs. Resolving this inherent contradiction remains a focal point of research even after more than 90 years of development in polymer physics.

Here, we propose a molecular strategy for constructing polymers using four-arm dynamic chemically coupled cross-linking units to establish an paradigm-shifting structure-property relationship where processability is improved with increasing cross-link density. The key to the structure design is the tetrafunctional cross-linker diaminoglyoxime (DAG), where two oxime and two amino groups can react with isocyanates to generate dynamic covalent oxime-carbamate bonds and dynamic covalent amidine-urea bonds rich in hydrogen bonds, thus constructing a cross-linking network integrated with triple dynamic bonds. At room temperature, the networks of higher cross-link densities demonstrated substantial enhancements in mechanical performance when compared to their counterparts with lower cross-link density. Meanwhile, networks with a high cross-link density have a high content of dynamic bonds. At elevated temperatures, the dynamic amidine-urea bond exhibits a catalytic effect on the dynamic oxime-carbamate bond, which notably enhances the dissociation of the network, leading to rapid relaxation of the material. Consequently, the modulus decrease rate with temperature outperforms all reported dynamic cross-linked polyurethanes. This approach enhances mechanical performance of the materials at ambient temperatures while reducing the solid-liquid transition temperature—a phenomenon rarely observed in the polymer field (*14*–*18*). Simultaneously enhancing cross-link density and processability of the resultant polymer subverts the conventional structure-property relationship. Overall, the design of chemically coupled dynamic cross-link site reconcile the long-standing contradiction between the mechanical and processing properties of polymers, emerging as a powerful principle for modulating material properties.

## RESULTS

### Design and synthesis of DAG polyurethane

**Fig. 1.**
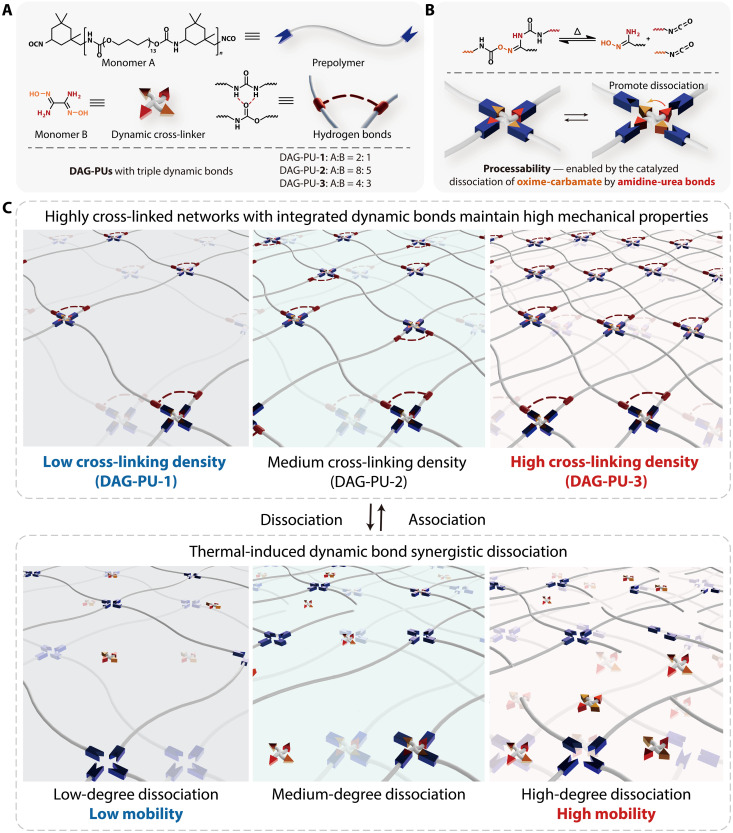
Structure design of DAG-PU elastomers for simultaneously improving processability and mechanical performance. (**A**) Chemical structures and cartoon representation of prepolymer monomer A and the cross-linker monomer B, as well as hydrogen bond unit. (**B**) Schematic illustration of the mechanism of the dynamic oxime-carbamate bonds and the dynamic amidine-urea bonds. (**C**) Schematic illustration of the polymer networks at room temperature and the dynamic properties at high temperatures.

DAG polyurethane (DAG-PU) elastomers were synthesized via one-pot polycondensation using polytetramethylene ether glycol (PTMEG), isophorone diisocyanate (IPDI), and DAG as raw materials and catalyst dibutyltin dilaurate (fig. S1 and table S1). The multiarm cross-linker DAG was the key to this elastomer. DAG is crucial in the design of DAG-PU, whose two oxime groups can form reversible dynamic oxime-carbamate bonds with isocyanates, while its two amino groups can form dynamic amidine-urea bonds, which provide many hydrogen bonds that dissipate energy during mechanical deformation and lead to high toughness. The simultaneous presence of multiple dynamic bonds gives it processability, while the relatively strong chemical cross-linking ensures its stable mechanical performance and structural stability (Fig. 1).

### Structural characterization and mechanical properties of DAG-PU

**Fig. 2.**
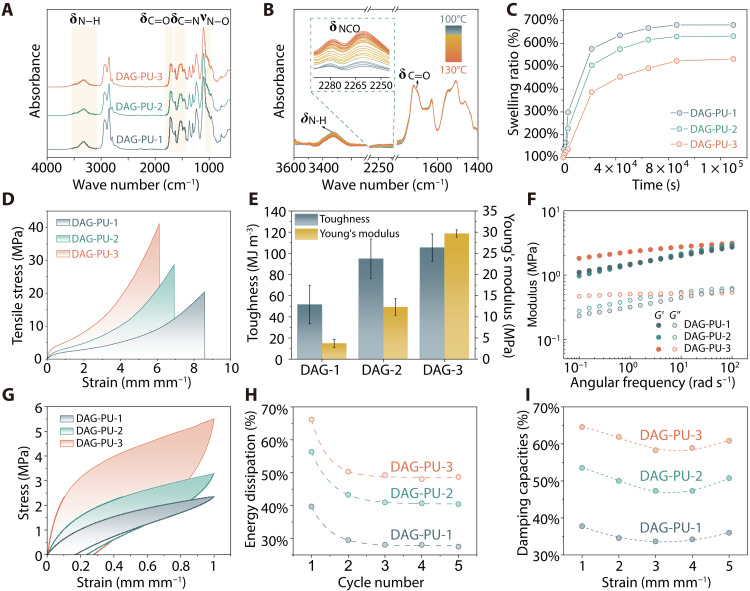
Structure and mechanical properties characterization of the DAG-PU elastomers. (**A**) FTIR spectra of DAG-PU-1, DAG-PU-2, and DAG-PU-3. (**B**) Temperature-variable FTIR spectra of DAG-PU-3 upon heating from 100° to 130°C in the regions of δ(C═O), δ(N─H), and δ(─N═C═O) (interval, 5°C). (**C**) Swelling ratio curves of DAG-PU-1, DAG-PU-2, and DAG-PU-3. (**D**) Stress-strain curves of DAG-PU-1, DAG-PU-2, and DAG-PU-3. (**E**) Toughness and Young’s modulus of DAG-PU-1, DAG-PU-2, and DAG-PU-3 were calculated from their stress-strain curves. (**F**) Frequency sweep rheological analysis of the DAG-PU-1, DAG-PU-2, and DAG-PU-3 in the range of 0.1 to 100 rad s^−1^. (**G**) Cyclic tensile tests of DAG-PU-1, DAG-PU-2, and DAG-PU-3 with an applied maximum strain of 100%. (**H**) The dissipated toughness ratio was calculated for DAG-PU-1, DAG-PU-2, and DAG-PU-3 within five successive loading-unloading tensile tests. (**I**) Damping capacities of DAG-PU-1, DAG-PU-2, and DAG-PU-3 calculated on the basis of their cyclic tensile tests.

The structures of the polymer networks were first investigated in the condensed state by Fourier transform infrared spectroscopy (FTIR) in the attenuated total reflection mode. As shown in Fig. 2A, the peak around 2275 cm^−1^ corresponding to the ─N═C═O groups disappeared completely in the polymer networks, which indicated that the IPDI and formed monomers with ─N═C═O groups were completely reacted. The peaks at 3329 and 1717 cm^−1^ were assigned as the stretching vibrations of N─H and C═O, respectively, which were consistent with the formation of urethane groups. At the same time, peaks at 1533 and 954 cm^−1^ are attributed to the stretching and bending vibrations of ─C═N─ and N─O groups, respectively, confirming the presence of DAG within the polymer network. Temperature-dependent in situ FTIR was used to elucidate the dynamic characteristics of DAG-PU. The results revealed the integrity of the dynamic bonds in DAG-PU at room temperature. In addition, a minor portion of free isocyanate (2275 cm^−1^) became detectable from oxime-carbamate and amidine-urea bonds upon elevating the temperature to 100°C (Fig. 2B). Beyond 130°C, evident dissociation of the carbamate and urea bonds within DAG-PU occurred. We then conducted a swelling test of the sample in tetrahydrofuran to confirm the formed network structure. After being soaked in tetrahydrofuran for 30 min, the size of the specimen increased markedly (Fig. 2C). In this case, the high cross-linking network in DAG-PU-3 will limit the movement of chain segments, which was the reason for its low expansion. In contrast, DAG-PU-1 with low cross-linking density had a higher swelling rate, which also proved our design.

The mechanical properties of DAG-PU elastomers with different cross-linking degrees were investigated via tensile tests. As shown in Fig. 2D, all samples exhibited typical rubber-like behavior in their stress-strain curves. The DAG-PU-3 had the highest Young’s modulus and strength due to its highest cross-linker content and highest hydrogen bond density. The Young’s modulus of DAG-PU-1, DAG-PU-2, and DAG-PU-3 were 3.8 ± 1.0, 12.3 ± 2.0, and 29.7 ± 0.9 MPa, respectively, while their tensile strength was 19.5 ± 1.6, 28.6 ± 8.1, and 45.0 ± 1.7 MPa, respectively. The fracture tensile strains decreased with the increase of cross-linking sites (*19*) and were 1039.7 ± 32.8, 717.3 ± 23.3, and 567.3 ± 37.8%, respectively (Fig. 2E).

The frequency sweep results of rheology tests were in line with those in tensile tests (Fig. 2F). The storage modulus of DAG-PU-3 was higher than that of DAG-PU-1 and DAG-PU-2, indicative of the stiffness of networks with higher cross-linking density. The microphase separation in DAG-PU-3 was more pronounced in the atomic force microscopy modulus mapping image compared to DAG-PU-1 (fig. S2), due to its higher cross-linking density. This conclusion was supported by small-angle x-ray scattering results (fig. S3), which indicated that the average distance between periodic hard domains in DAG-PU-3, calculated using Bragg’s law, was approximately 8.2 nm, whereas DAG-PU-1 did not exhibit notable microphase separation in small-angle x-ray scattering analysis. Furthermore, DAG-PU-3 showed a lower phase shift in the phase images compared to DAG-PU-1, indicating increased elasticity (fig. S4) (*20*). This finding supported the notion of stronger hydrogen bonding interactions within DAG-PU-3, consistent with the expected behavior based on structural modifications.

Subsequently, the cyclic stress-strain tests were conducted over a maximum strain range spanning from 100 to 500% (Fig. 2G and figs. S5 to S7), and the quantitative analysis of energy dissipation was summarized in Fig. 2H. Notably, all elastomers manifested considerable hysteresis, with the magnitude of the hysteresis loops escalating as the degree of cross-linking increased. This escalation was primarily ascribed to the dissociation of the prevalent hydrogen bonding stemming from the polyurethane segments. The damping capability, defined as the ratio of energy dissipation to input energy, has been summarized in Fig. 2I based on calculations derived from cyclic tensile tests (figs. S8 to S10). Variations in damping capacity may be associated with the impact of cross-linking on energy dissipation mechanisms. Results indicated a decreasing trend in damping capability with increasing strain levels imposed on specimens of varying cross-linking densities, which might stem from the intrinsic propensity of chemically cross-linked networks to recover their original dimensions at high strains predominantly through elastic deformation (*21*).

### Thermal properties, self-healing, and processability of DAG-PU

**Fig. 3.**
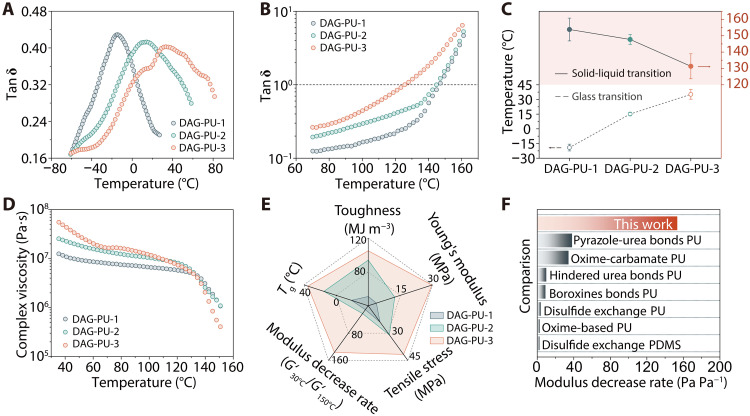
Performance of the DAG-PU elastomer. (**A**) Curves of the DAG-PU-1, DAG-PU-2, and DAG-PU-3 using DMA. (**B**) Tan δ of DAG-PU-1, DAG-PU-2, and DAG-PU-3 using rheology test. The solid-liquid transition occurs at approximately 150° to 130°C. (**C**) Summary of data on glass transition temperature and solid-liquid transition temperature for DAG-PU-1, DAG-PU-2, and DAG-PU-3. (**D**) Viscosity-temperature curves of DAG-PU-1, DAG-PU-2, and DAG-PU-3. (**E**) Comparison of the mechanical properties and processability of DAG-PU-1, DAG-PU-2, and DAG-PU-3. (**F**) A comparison of the modulus decrease rate for DAG-PU and other representative dynamic cross-linking polyurethanes. PDMS, polydimethylsiloxane.

The advantages of tetrafunctional cross-links in ensuring the mechanical properties of materials have been confirmed, and then we investigated their enhancement of processing performance. As shown in Fig. 3A and figs. S11 to S13, the dimethylamine (DMA) results revealed that all the samples had two distinguishing glass transition temperatures (*T*_g_). The transition at approximately −60°C observed for DAG-PU was attributed to the relaxation of the soft PTMEG segments, which was independent of the content of stiff cross-links. Note that DAG-PU-1 to DAG-PU-3 also exhibited a second *T*_g_, with measured values ranging from −15.2° to 33.8°C. The upward trend in *T*_g_ values could be attributed to the increased incorporation of hard segments, leading to a denser network structure and consequently impeding the mobility of polymer chain segments.

To further understand the temperature dependence of viscoelasticity, storage modulus (*G*′) and loss modulus (*G*″) were investigated in an extended temperature sweep and plotted in figs. S14 to S16. The temperature at which the tangent of the loss factor (tan δ) reaches unity represents the transition temperature (*T*_f_), indicating the shift from a rubbery state to a viscous state. Conventionally, this temperature can be approximated as the lower boundary of the reprocessing temperature range of the materials. A decreasing trend was observed for the *T*_f_ at which viscous responded dominate viscoelastic behavior with values of 153.8°, 147.7°, and 131.3°C recorded for DAG-PU-1, DAG-PU-2, and DAG-PU-3 correspondingly (Fig. 3B), indicative of accelerated relaxation with increased dynamic bond concentration in DAG-PU. Below *T*_f_, the elastic plateau was inconspicuous for the DAG-PU-3 but was obvious for the DAG-PU-1, which implied that DAG-PU-3 had a more dynamic network than the DAG-PU-1 and DAG-PU-2. Above *T*_f,_ both *G*′ and *G*″ decreased with increasing temperature for all three samples. The specific data on the glass transition temperature (*T*_g_) and solid-liquid transition temperature (*T*_f_) of DAG-PU-1, DAG-PU-2, and DAG-PU-3 have been summarized in Fig. 3C. Figure 3D illustrated the alternating viscosity change trends of DAG-PU-1, DAG-PU-2, and DAG-PU-3. A marked transition in complex viscosity occurred near the solid-liquid transition point. DAG-PU-3 exhibited higher cross-linking density and complex viscosity at low temperatures. However, upon heating to the dissociation temperature, the high content of dynamic bonds in DAG-PU-3 led to a rapid collapse of the polymer network through dissociation, breaking into the small molecular chain, resulting in a substantial decrease in complex viscosity for DAG-PU-3 compared to DAG-PU-1. To further characterize the impact of DAG content on the material’s processability, we conducted melt index tests on three samples, with the results presented in table S2. The results showed that the melt index increased from 12.5 g 10 min^−1^ for DAG-PU-1 to 26.3 g 10 min^−1^ for DAG-PU-3. This increase was primarily attributed to the higher DAG content, which resulted in a greater number of short chains in the melt, thereby enhancing flowability. For a clearer comparison, we presented the representative mechanical performance parameters including Young’s modulus, maximum stress, toughness, *T*_g_, and modulus decrease rate of all the samples by a radar chart in Fig. 3E.

The results showed that further increasing the cross-linking density led to a sharp rise in *T*_g_, from 33.8°C for DAG-PU-3 to 61.0°C for DAG-PU-4 and 66.0°C for DAG-PU-5 (fig. S17, A and B). Similarly, the Young’s modulus increased notably, from 29.7 ± 0.9 MPa for DAG-PU-3 to 85.7 ± 12.9 MPa for DAG-PU-4 and 195.6 ± 13.6 MPa for DAG-PU-5. The minor changes in toughness could be attributed to the highly cross-linked network, which limited elongation at break (591.0 ± 38.9% for DAG-PU-4 and 521.3 ± 24.6% for DAG-PU-5), while the tensile strength remains essentially unchanged (42.1 ± 0.9 MPa for DAG-PU-4 and 42.0 ± 2.3 MPa for DAG-PU-5) (fig. S17C). Because of the notably reduced segmental mobility, DAG-PU-4 and DAG-PU-5 maintained a stable storage modulus across a wide temperature range, without a notable drop in the temperature sweep (fig. S17, D and E). This made DAG-PU-3 the formulation with the best overall performance (fig. S17F). Notably, even when the cross-linker content was doubled, the solid-liquid transition temperatures of DAG-PU-4 and DAG-PU-5 (155.6° and 158.9°C, respectively) did not show a substantial increase compared to DAG-PU-1 (153.8°C). These results highlighted the advantages of high dynamic cross-link density in ensuring the processability properties of DAG-PU. Figure 3F summarized the ratio of storage moduli for all readily processable chemically cross-linked polyurethanes at temperatures of 30° and 150°C. At a temperature differential Δ*T* of 120°C (spanning from 30° to 150°C), the soft-to-hard transition capability, denoted as σ (defined as *G*′_max_/*G′*_min_) (*22*), yields a substantial modulus decrease of 15,330% for DAG-PU-3. Such a marked alteration in storage modulus over a narrow temperature interval has never before been documented in dynamically cross-linked polyurethane (*6*, *17*, *23*–*28*).

This result provided intuitive evidence for the enhanced thermal processing performance of materials with a high cross-linking degree. Thermogravimetric analysis was conducted to assess the thermal stability of the polymer network. All polymer formulations displayed analogous thermal decomposition behavior. Within these, the temperature at which a 5% weight loss occurred diminishes with rising DAG component content, as depicted in the thermogravimetric analysis outcomes (fig. S18). In addition, the presence of dynamic bonds facilitated network dissociation and consequently accelerated thermal decomposition. Subsequently, the self-healing performance of DAG-PU was characterized, with particular focus on DAG-PU-3, which exhibited the most balanced properties. As recorded by optical microscopy, the scratch on the DAG-PU-3 film could be repaired after 1 hour of healing at 80°C (fig. S19). Furthermore, the specimen was cut into two pieces and put back into contact with gentle pressure. Then, the samples were placed in an oven to heal at different times under 100°C to quantitatively evaluate the self-healing ability of the DAG-PU-3 by tensile tests. The repairing efficiency was defined as the ratio of the fracture strain of the healed film divided by that of the uncut film. As shown in fig. S20A, the self-healing efficiency went up to 90% after 24 hours, along with a high stretchability of 650%. In addition to the elongation, other mechanical properties such as toughness and tensile strength were also recovered upon increasing the healing time (fig. S20B). The processability of DAG-PU-3 was studied by the cyclic remolding experiment. In fig. S21A, we depicted the reprocessing process of the DAG-PU-3 sample, in which the mechanism of the dynamic bonds activated by heating was highlighted as well. The original specimen of the DAG-PU-3 was cut into small fragments and reprocessed by compression molding with a pressure of 5 MPa for 10 min under 110°C to obtain a remolded specimen. Distinct from self-healing properties, processability imposed a more stringent requirement on the dynamic nature of the cross-links (*29*). Such a procedure was repeated three times, as shown in fig. S21B. The virgin and reformed DAG-PU-3 exhibited almost the same FTIR spectra, indicating the retention of chemical integrity after reprocessing three times. Moreover, we performed the tensile tests and rheological temperature sweep of the reprocessed DAG-PU-3 and demonstrated its remarkable reprocessability in terms of the maintained mechanical performance including the storage modulus and toughness (figs. S21, C and D, and S23). The decent reprocessable performance of DAG-PU-3 implied favorable dynamic properties of tetrafunctional cross-linkers. Conventionally, enhanced robustness and strength correlate with diminished dynamicity, posing a pervasive challenge in polymer design (*30*). The above results demonstrated that the inherent tradeoff between robustness and dynamics could be effectively addressed by integrating dynamic bonds into the cross-linking sites.

### The structure-property relationships of the DAG-PU

**Fig. 4.**
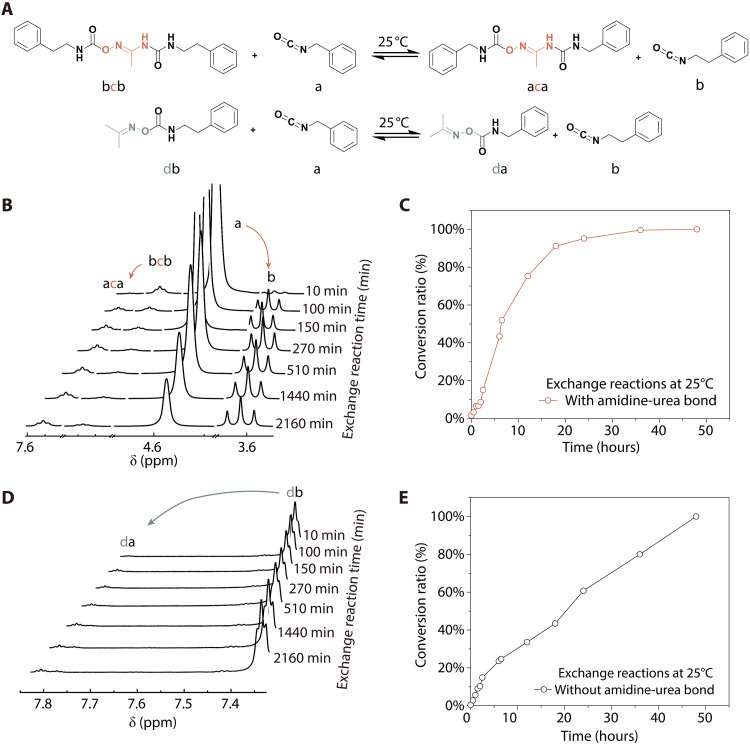
Model study of coupled multiple dynamic bonds. (**A**) Exchange reaction between model compounds **bcb** or **db** and **a** produced **aca** or **da** and **b** at 25°C. (**B**) Real-time ^1^H NMR spectra of the mixture of **bcb** and **a**. (**C**) Conversion of compound **aca** with amidine-urea bond [conversion ratio = (**aca**_0_ − **aca***_t_*) / (**aca**_0_ − **aca**_eq_); **aca**_0_, initial concentration of **aca**; **aca***_t_*, concentration of **aca** at time *t*; **aca**_eq_, equilibrium concentration of **aca**]. (**D**) Real-time ^1^H NMR spectra of the mixture of **db** and **a**. (**E**) Conversion of compound **da** without amidine-urea bond.

We conducted ^1^H nuclear magnetic resonance (NMR) and rheological experiment spectroscopy analysis in this section to gain a thorough understanding of the structure-property relationships of our DAG-PU. To investigate the reversibility of DAG units, we synthesized model compounds **bcb** and **db**. **bcb** was obtained by reacting *N*-hydroxyethylamine with phenyl isocyanate, while **db**, lacking the amidine-urea bond, was synthesized through the reaction of acetoxime with phenyl isocyanate (Fig. 4A). Subsequently, we mixed these two model compounds with benzyl isocyanate (compound **a**) in deuterated dimethyl sulfoxide-d_6_ (figs. S24 to S34). The ^1^H NMR spectrum of the compounds **bcb** and **a** mixture showed proton signals of nitrogen atoms at 5.88 and 7.07 parts per million (ppm), respectively (Fig. 4B). With time, two new proton signals at 7.55 and 3.58 ppm corresponding to the aforementioned methylene group near the nitrogen atom in products compound **aca** and phenethyl isocyanate (compound **b**) appeared, indicating the occurrence of an exchange reaction. It took nearly 36 hours to reach equilibrium at 25°C (Fig. 4C), whereas the exchange reaction without the amidine-urea bond did not reach equilibrium after 50 hours (Fig. 4, D and E). Hence, it could be inferred that the facilitative effect of the dual dynamic bonds within the DAG moiety leads to enhanced reversibility of the oxime-carbamate compared to oximes without the amidine-urea bond (*31*). The in situ ^1^H NMR spectrum of compound **bcb** reveals its dissociation reaction (fig. S35). First, the amidine-urea and oxime-carbamate units in compound **bcb** dissociated into isocyanate groups in compound **a** and amino and oxime groups in compound **c**. Compound **c** reacted with the isocyanate groups in compound **a** to form the amidine-urea and oxime-carbamate units in compound **aca**. These experiments pioneer the demonstration of the reversibility of amidine-urea bonds, thereby enhancing dynamic reconfiguration of DAG-PU network.

To correlate catalytic behavior with improved processability, we designed a polymer network containing dynamic oxime-carbamate bonds and used pentaerythritol as a tetrafunctional cross-linker, with the dynamic bond content and cross-linking density comparable to those of DAG-PU-3 (table S3 and fig. S36A). Uniaxial tensile tests revealed that control-PU has a strength of 17.6 ± 1.2 MPa and a toughness of 52.6 ± 1.3 MJ m^−3^, both notably lower than those of DAG-PU-3 (fig. S36B). This discrepancy might be attributed to the presence of urea bonds in DAG-PU-3, which provided additional hydrogen bonding. DMA showed that the glass transition temperature (*T*_g_) of control-PU was 65.5°C, considerably higher than the 33.8°C observed for DAG-PU-3. Temperature sweep results indicated no solid-liquid transition at 180°C for control-PU. This result not only confirmed the catalytic effect of the amidine-urea bond on the oxime-carbamate bond observed in small-molecule model experiments but also demonstrated that this catalytic effect facilitated network dissociation at high temperatures, thereby enhancing processability.

**Fig. 5.**
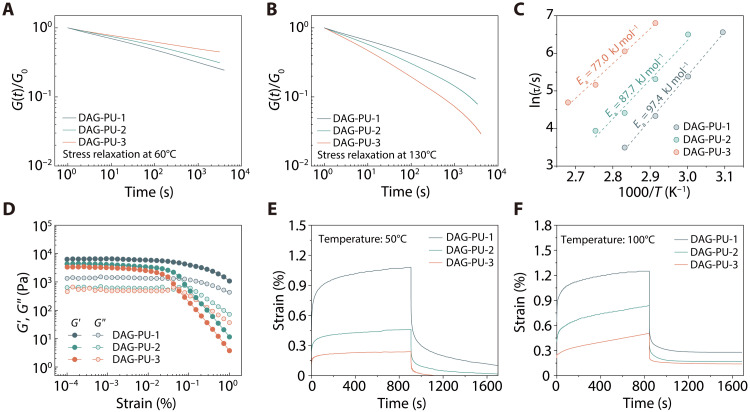
Insights into the structure-property relationships of DAG-PU elastomers. (**A**) Normalized stress relaxation curves of DAG-PU-1, DAG-PU-2, and DAG-PU-3 were measured at 60°C, respectively. (**B**) Normalized stress relaxation curves of DAG-PU-1, DAG-PU-2, and DAG-PU-3 were measured at 130°C, respectively. (**C**) The activation energies of DAG-PU-1 to DAG-PU-3 were calculated at varying temperatures according to the Arrhenius equation, thereby characterizing their respective relaxation times. (**D**) Strain sweep rheological analysis of DAG-PU-1, DAG-PU-2, and DAG-PU-3 at a constant angular frequency of 1.0 rad s^−1^. (**E**) Creep-recovery curves of DAG-PU-1, DAG-PU-2, and DAG-PU-3 with the same stress at 50°C and (**F**) 100°C.

Stress relaxation and creep experiments can also yield valuable structural information regarding the network. Bond dissociation in processable covalent network polymers results in stress relaxation, and the scaling relationship of characteristic relaxation times (τ*) with temperature is an important aspect of describing their rheological behavior. As depicted in Fig. 5A, minimal relaxation was observed among the three sample groups. Notably, DAG-PU-3 did not relax after 3000 s at 60°C, whereas DAG-PU-1 and DAG-PU-2 underwent slightly faster stress relaxation behaviors. Subsequently, the same stress was applied to DAG-PU-1, DAG-PU-2, and DAG-PU-3 at 130°C where distinct changes were noted (Fig. 5B). Results indicated that DAG-PU-3 rapidly relaxed within 100 s, which was diametrically opposite results compared to those obtained at 60°C. As mentioned earlier, at 60°C, stress maintenance was commendable until the onset of substantial bond dissociation, followed by accelerated stress relaxation upon elevating temperatures. The traditional 1/*e* method using stress relaxation experiments was first applied to determine 𝜏 as the time when the normalized stress decreases to 36.8% (1/*e*) of the initial value. The ln(𝜏) showed a remarkable linear relationship versus the 1000/*T* (Fig. 5C and figs. S37 to S39), and their slopes give the apparent activation energies of 97.4, 87.7, and 77.0 kJ mol^−1^ for the DAG-PU-1, DAG-PU-2, and DAG-PU-3, respectively. Typically, an increase in the number of dynamic bonds heightens the sensitivity of dynamic exchange to temperature. Conversely, augmenting cross-link density tends to reduce the temperature sensitivity of processable covalent network polymers. In this work, we simultaneously augmented the number of dynamic bonds and cross-linking sites, with the process further enhanced by the catalytic influence of the amidine-urea bond on the oxime-carbamate bond. The results revealed a synergistic interaction between these two elements within a certain range of cross-linking densities, leading to a reduction in activation energy (*32*). This trend became more pronounced with an increase in DAG content. Since this activation energy is associated with the dissociation of dynamic cross-links in DAG-PU, the lower activation energy of DAG-PU-3 suggests that its dynamic cross-links could be more easily dissociated with increasing temperature, thereby improving its processability. We studied the temperature-induced structural changes of the three samples using strain sweep tests. At the onset, under low–shear strain conditions, stability prevailed with negligible reduction in *G*′ and *G*″, corresponding to the linear viscoelastic domain of DAG-PU. Variations in *G*′ and *G*″ with escalating oscillation amplitudes serve as indicators of the network’s coherence. Figure 5D illustrated that at a 5.25% oscillatory strain, the initial crossover of *G*′ and *G*″ for DAG-PU-3 occurred, implying network disruption induced by extensive dynamic bond dissociation. On the other hand, under the same strain conditions, the *G*′ and *G*″ of DAG-PU-1 showed smaller variations over larger oscillation amplitudes because of its low density of dynamic bonds, and the network could be maintained. As revealed by rheological experiments, at temperatures below extensive dynamic cross-linking dissociation, the networks remained resilient even under large imposed strain. Conversely, once notable bond dissociation occurred, smaller strains could readily compromise the networks. To verify the stability of the polymer before network dissociation, we conducted creep and recovery experiments on DAG-PU-1, DAG-PU-2, and DAG-PU-3 at 50° and 100°C under an applied load of 800 Pa, respectively. During the creep process at 50°C, all three samples displayed a negligible increase in strain immediately, followed by reaching a creep equilibrium. The strain of DAG-PU-3 at 50°C was nearly fully recovered less than 300 s after stress removal (Fig. 5E). These results indicated that the DAG-PU-3 dynamic chemistry is extremely slow at 50°C allowing both creep arrest and full or nearly full strain recovery at this temperature. Furthermore, in the recovery test, DAG-PU-3 exhibited lower residual strain and a superior recovery rate compared to DAG-PU-1, which could be attributed to the higher cross-link density. DAG-PU-3 also maintained its excellent creep resistance at 100°C; the network exhibits a creep strain of only 0.15%. We noted that the oxime-carbamate bonds and amidine-urea bonds in DAG-PU have been shown to undergo perceptible dissociation reactions at 100°C (Fig. 5F). Thus, we expected that DAG-PU dynamic chemistry was active but at very low levels during 100°C the creep test. This was also confirmed by the creep-recovery behavior of the network at 100°C as the strain was incompletely recovered 300 s after stress removal. However, given the negligible creep strain and the low residual strain, we believe that the rate of triple dynamic bond dissociation is sufficiently low to nearly arrest creep at 100°C. This study provides a comparative investigation of the decoupling of mechanical properties and thermal processing characteristics of dynamic polymer networks through topology design. We anticipate that the fundamental insights gained from our research will guide the development of high-performance, easily processable polymers through topology network design strategies.

## DISCUSSION

In summary, we present a molecular design strategy based on chemically coupled four-arm dynamic sites, which overcomes the enduring contradiction between the tensile property and processability of polymers. The resulting DAG-PU elastomers exhibit a paradigm-shifting structure-property relationship, where polymers with higher cross-link densities exhibit better processing capabilities. These elastomers exhibit remarkable soft-to-hard switching capability, manifested in a swift modulus reduction over a predefined temperature range of 120°C, measured at 153.3, notably exceeding all recently reported processable cross-linked polyurethanes by more than fourfold. The superior mechanical properties of DAG-PU stem from enhanced cross-link densities and microphase separation structures promoted by amidine-urea segment-enriched hydrogen bonding, conferring excellent creep resistance and damping behavior, thereby enhancing durability under practical usage conditions. The outstanding processability is attributed to the combined design of the integration of multiple dynamic bonds within the cross-linking sites and the catalytic effect of amidine-urea bonds on oxime-carbamate bonds. Furthermore, the DAG-PU elastomers enable thermally triggered self-healing and reshaping, restoring their mechanical properties, prolonging service life, and minimizing material waste. We contend that this molecular design, integrating electronic effects and topological structures, introduces a potent new paradigm for modulating material characteristics.

## MATERIALS AND METHODS

### Materials

PTMEG (Mn = ~1000 g mol^−1^), IPDI (99%), and dibutyltin dilaurate (DBTDL; 95%) were purchased from Aladdin. The dimethyl sulfoxide-d_6_ and phenethyl isocyanate were purchased from ADAMAS-BETA. Propyl isocyanate was obtained from 9dingchem. DAG (95%) and *N*-hydroxyacetamidine (95%) were purchased from Bide Pharmatech Ltd. *N*,*N*-dimethylformamide (DMF; 99.8%) were purchased from J&K Chemical. Phenethyl isocyanate was purchased from Adamas (China). All reagents were used as received without further purification unless otherwise noted.

### Synthesis of DAG-PU and control-PU

#### Diaminoglyoxime polyurethane

Taking DAG-PU-3 as an example, PTMEG (4 g, 4 mmol) was poured into the reactor and dried at 110°C for 120 min in a vacuum. Here, the reactor was then cooled to 80°C. DAG (0.3543 g, 3 mmol) were dissolved in 20 ml of DMF, and the mixture was then added to the cooled reactor containing dried PTMG. DBTDL (0.2 wt %, 0.02 g) and IPDI (2.2228 g, 10 mmol) were sequentially added to the mixture. The reaction occurred at 80°C for 15 hours under a nitrogen atmosphere. Then, the reaction mixture was poured into a polytetrafluoroethylene mold and dried under vacuum at 80°C for approximately 24 hours and then at 100°C for 6 hours to ensure complete removal of DMF.

#### Control-PU

PTMEG (4 g, 4 mmol) was poured into the reactor and dried at 110°C for 120 min in a vacuum. Here, the reactor was then cooled to 40°C. Dimethylglyoxime (0.1682 g, 6 mmol) were dissolved in 8 ml of DMF, and the mixture was then added to the cooled reactor containing dried PTMG. DBTDL (0.5 wt %, 0.04 g), pentaerythritol (0.4085 g, 3 mmol), and IPDI (3.5565 g, 16 mmol) were sequentially added to the mixture. The reaction occurred at 80°C for 15 hours under a nitrogen atmosphere. Then, the reaction mixture was poured into a polytetrafluoroethylene mold and dried under vacuum at 80°C for approximately 24 hours and then at 100°C for 6 hours to ensure complete removal of DMF.
