# Supplementary material for: Dynamic cross-linked topological network reconciles the longstanding contradictory properties of polymers
Source: Sci Adv. 2025 Mar 19;11(12):eadt0825. doi: 10.1126/sciadv.adt0825 (PMC11922047; doi:10.1126/sciadv.adt0825)
Supplement: Supplementary file 1 — Supplementary Text Figs. S1 to S39 Tables S1 to S3 [file sciadv.adt0825_sm.pdf]

Science Advances
AAAS

Supplementary Materials for

Dynamic cross-linked topological network reconciles the longstanding contradictory properties of polymers

Zekai Wu *et al.*

Corresponding author: Zhengwei You, zyou@dhu.edu.cn

Sci. Adv. **11**, eadt0825 (2025)
DOI: 10.1126/sciadv.adt0825

This PDF file includes:

Supplementary Text
Figs. S1 to S39
Tables S1 to S3

Supplementary Text

1. *General methods*

General Characterization Information: All tests were performed at room temperature unless otherwise noted. Nuclear magnetic resonance (NMR) spectra were recorded with a Bruker Avance 600 spectrophotometer with the use of the deuterated solvent as the lock and the residual solvent or TMS as the internal reference.

FTIR spectra were recorded on a Nicolet 8700 spectrometer (Thermo Electron Corporation, USA). Attenuated total reflectance Fourier transform infrared (ATR-FTIR) spectra were recorded on a ThermoFisher Scientific Nicolet iS50 spectrometer with an ATR accessory.

The two-dimensional (2D) small angle X-ray scattering (SAXS) patterns were acquired using a Xeuss3.0SAXS/WAXS. The detector model is Eiger2R1M, the pixel side length is 75 microns, and the copper target 8.05KeVX-ray is used, with a wavelength of 1.54189 angstroms. Each 2D SAXS experiment was conducted by exposing the sample to the X-ray source for 10 min. The wavelength of the X-ray radiation was 0.154 nm, and the sample-to-detector distance was 1200 mm. The one-dimensional (1D) SAXS curves were obtained using DIFFRAC.SAXS software. The periodicity (L) was calculated according to Bragg's Law:

$$L = \frac{2\pi}{q_{max}}$$

where $q_{\max}$ corresponds to the peak position of the 1D SAXS curve.

The surface morphology of specimens was investigated using */Dimension FastScan/lcon Atomic force microscopy (AFM) with an aluminum reflex-coated silicon cantilever probe (HQ: NSC19/Al BS).

The glass transition temperature is obtained by dynamic thermodynamic analysis on a DMA1 (METTLER TOLEDO). Dynamic mechanical analyzer. Rectangular samples (ca. 1 mm (T) × 5 mm (W) × 10 mm (L)) were tested at a frequency of 1 Hz and a strain of 0.1%. Heating ramps of 5 °C min^{-1} were applied from -120 to 80 °C. T_g values were calculated from the maximum value of tan Delta.

Thermogravimetric analysis (TGA) was used to study the dynamic thermal stability utilizing a thermogravimetric analyzer (Libra/209F1). The synthesized copolymers were evaluated to 600 °C using a heating rate of 10 °C·min^{-1} under a nitrogen atmosphere.

Temperature sweep tests were performed on an Anton Paar MCR702 rheometer equipped with a 25 mm solid-mode rotor. The tests applied a 0.5% strain at a frequency of 1 Hz to samples that were 1 mm thick.

The normalized stress-relaxation experiments were performed in a strain control (10% strain) mode. The relaxation modulus (G) was normalized by the initial value (G_0). E_a represents the activation energy, τ_0 represents the characteristic relaxation time at 1/e, R is the gas constant, T is the temperature relaxation test, and τ(T) corresponds to the time when the modulus relaxes to 1/e. E_a can be calculated by multiplying the slope obtained by plotting ln(τ)-1000/T by the constant R. The characteristic relaxation time (τ*) was defined as the time required for $G/G_0 = 1/e$ with exponential decay function: $G(t) = G_0 \exp(-t/\tau^*)$.

The mechanical properties of the samples were assessed by an MTS E42 tensile machine equipped with a 100 N load cell. The uniaxial tensile tests were carried out while the crosshead was adjusted at 50 mm·min^{-1}. Rectangular tensile bars (1 mm (T) × 3 mm (W) × 20 mm (L)) cut from large films were used. The deflection rate of uniaxial tensile measurements was 50 mm min^{-1}. At least three specimens were tested and averaged for each sample. If not specified, cycle tensile tests were performed at a tensile rate of 50 mm·min^{-1} and a recovery rate of 50 mm min^{-1}. Energy dissipation was calculated by integrating the area encompassed by the cyclic tensile curves. Damping capacity was defined as the ratio of the dissipated energy (the area encompassed by the loading and unloading curves) to the loading energy (the area encompassed by the loading curve).

Self-healing tests were evaluated mainly by scratch recovery and restoration of mechanical properties for various periods. Scratch recovery tests were performed by scratching films with a blade. The changes of the scratch-width track were using an optical microscope (MDA2000, Future Optics).

Cut DAG-PUs into small pieces and put them into a fluid flow rate meter (MFI-1211, JJ-TEST, China) to determine the melt index (MI). Experiment condition was set as follows: the load was 2160 g, and the temperature was 160 °C. Samples were collected every 60 s for 10 min and were measured to calculate the melt index.

2. *Syntheses of DAG-PU*

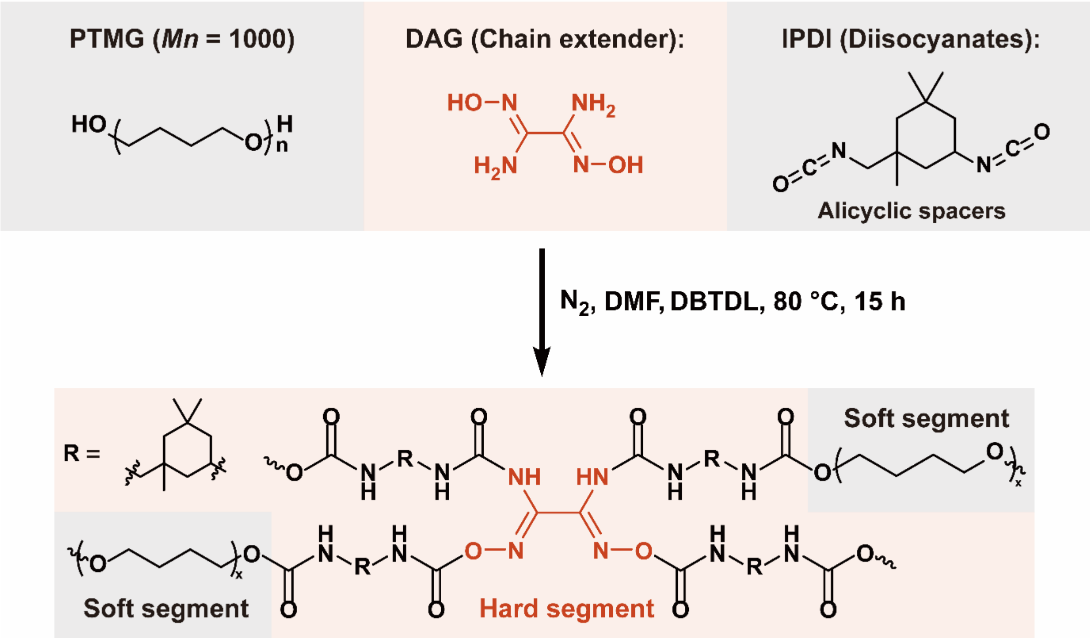

Fig. S1 Synthetic route of the DAG-PU network

Table S1 The composition of all samples

Sample	PTMG-1000	IPDI	DAG	DBTDL
DAG-PU-**1**	0.0040 mol	0.0080 mol	0.0020 mol	0.02g
DAG-PU-**2**	0.0040 mol	0.0090 mol	0.0025 mol	0.02g
DAG-PU-**3**	0.0040 mol	0.0100 mol	0.0030 mol	0.02g
DAG-PU-**4**	0.0040 mol	0.011 mol	0.0035 mol	0.02g
DAG-PU-**5**	0.0040 mol	0.012 mol	0.004 mol	0.02g

3. Characterization for DAG-PU-1, DAG-PU-2 and DAG-PU-3

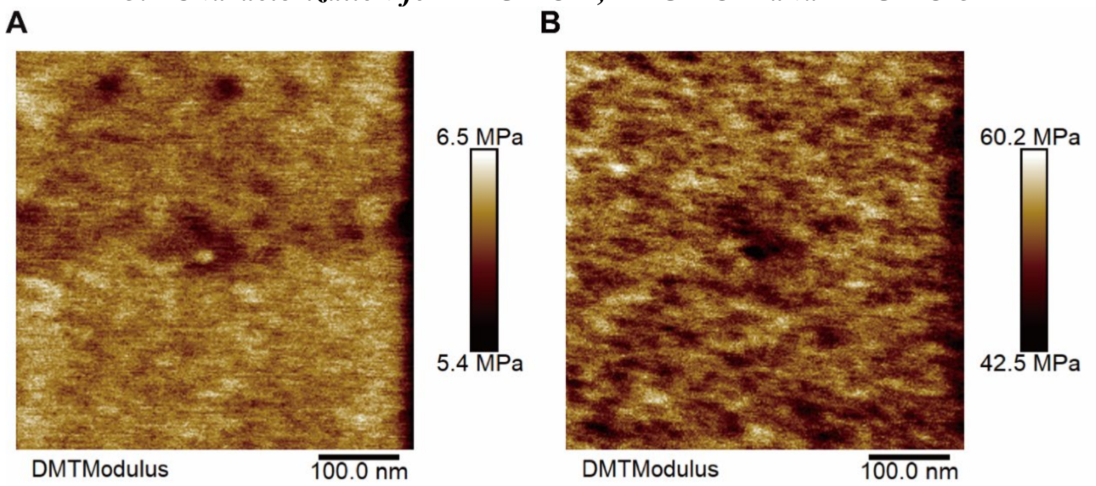

Fig. S2 AFM modulus mapping of (A) DAG-PU-1 and (B) DAG-PU-3.

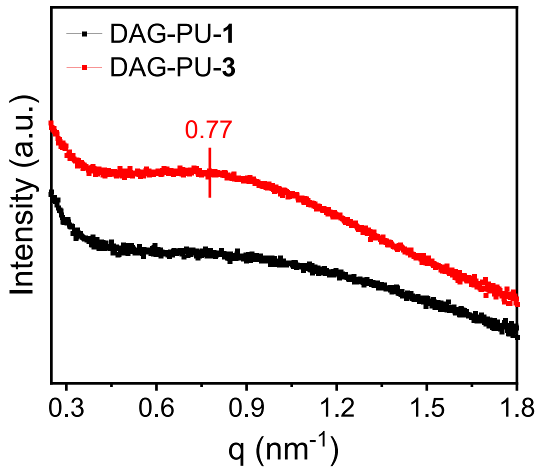

Fig. S3 1D SAXS profiles of the DAG-PU-1 and DAG-PU-3 elastomers.

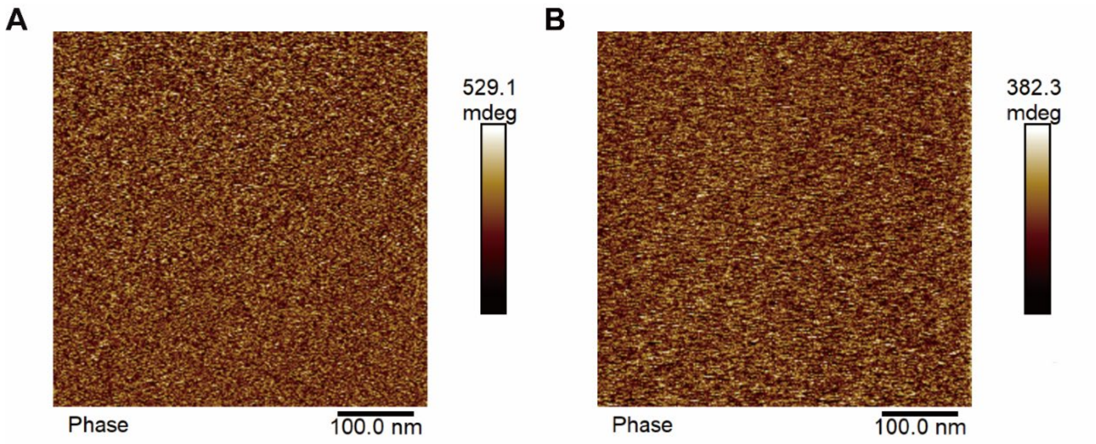

Fig. S4 AFM phase images of (A) DAG-PU-1 and (B) DAG-PU-3.

4. Mechanical properties of DAG-PU-1, DAG-PU-2 and DAG-PU-3

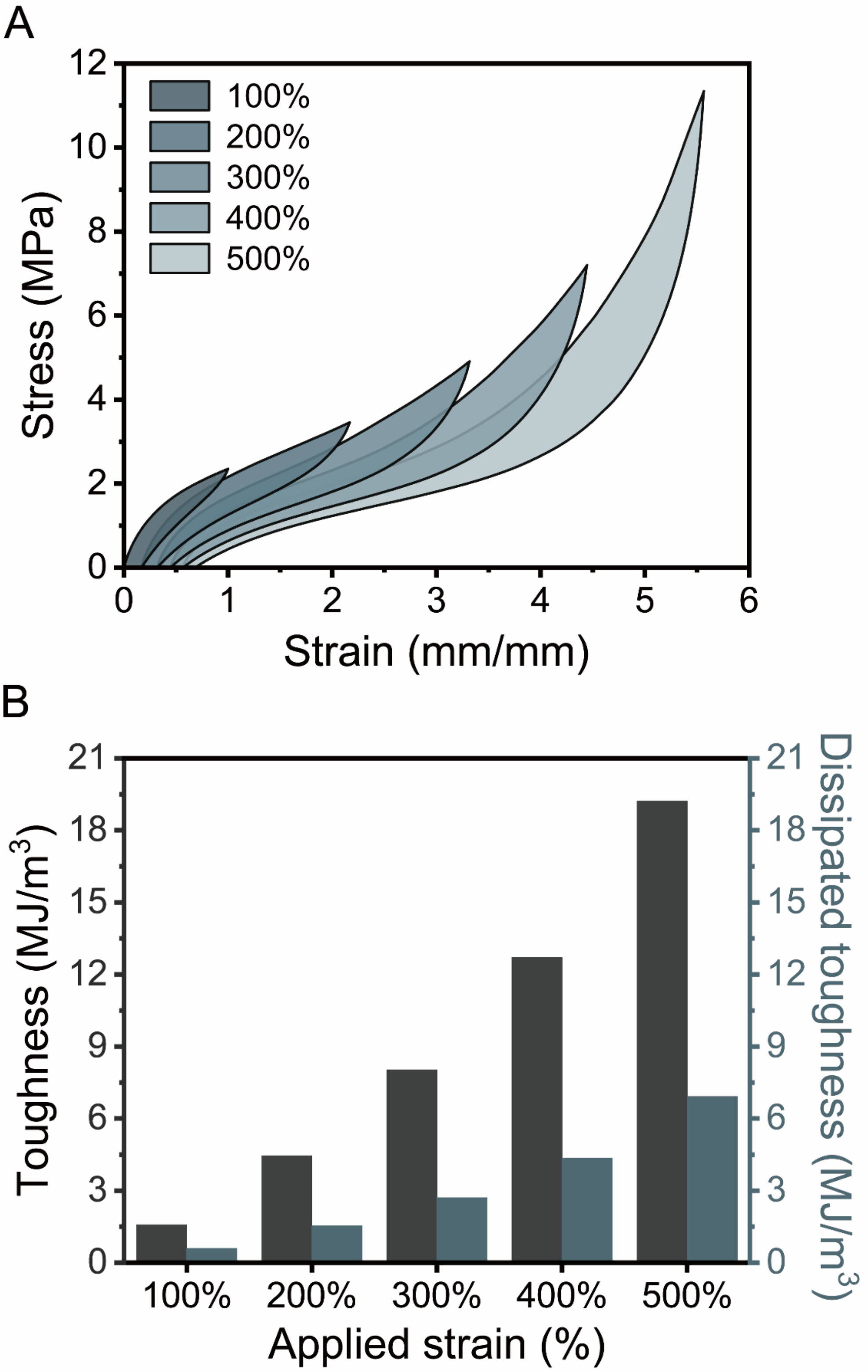

Fig. S5 **The cyclic tensile tests with gradually larger strains of DAG-PU-1** (A) Continuous cyclic tensile curves under different maximum strains. (B) The total and dissipated toughness calculated for DAG-PU-1 within five cyclic stress-strain tests in the maximum strain range from 100% to 500%.

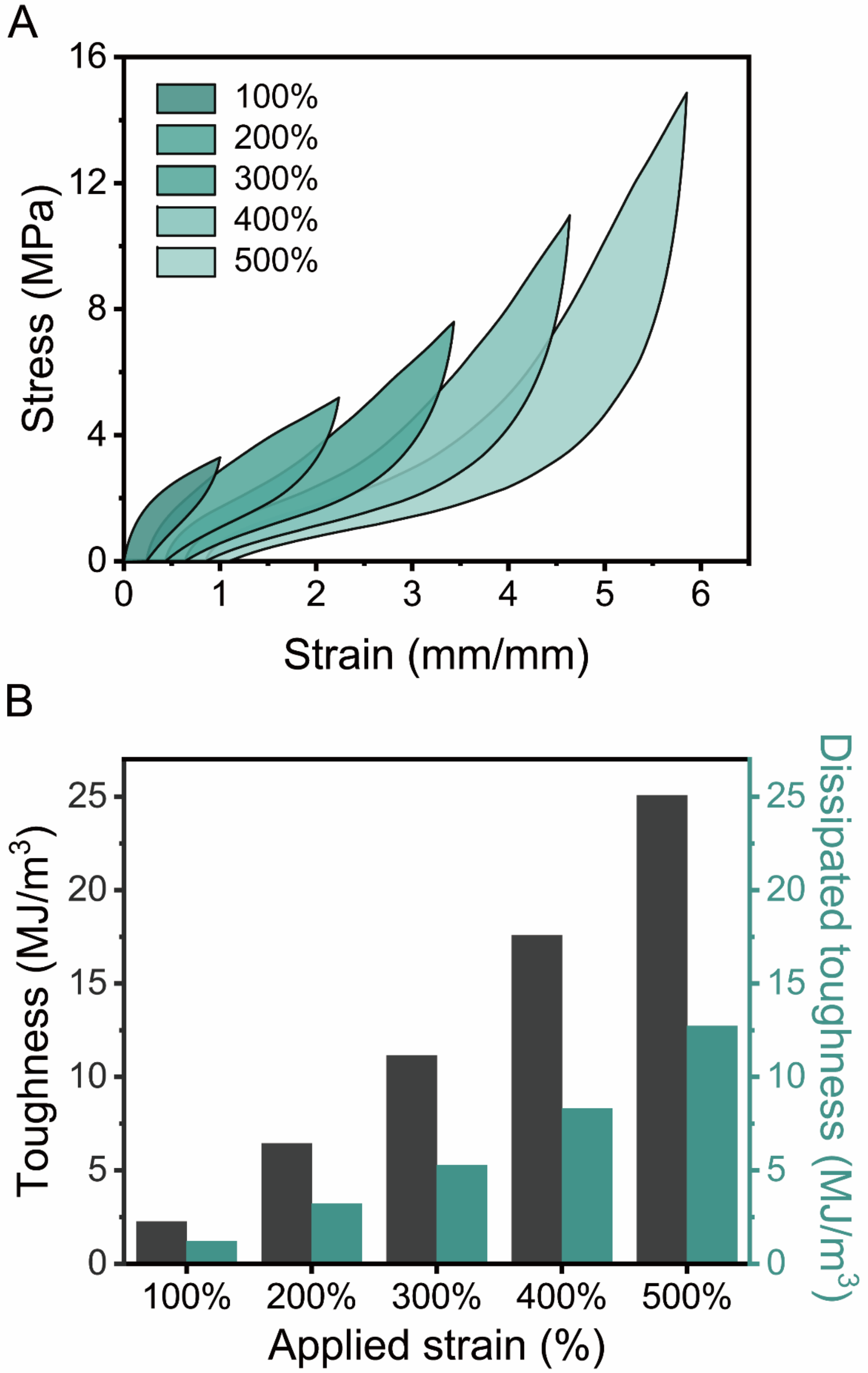

Fig. S6 **The cyclic tensile tests with gradually larger strains of DAG-PU-2** (A) Continuous cyclic tensile curves under different maximum strains. (B) The total and dissipated toughness calculated for DAG-PU-2 within five cyclic stress-strain tests in the maximum strain range from 100% to 500%.

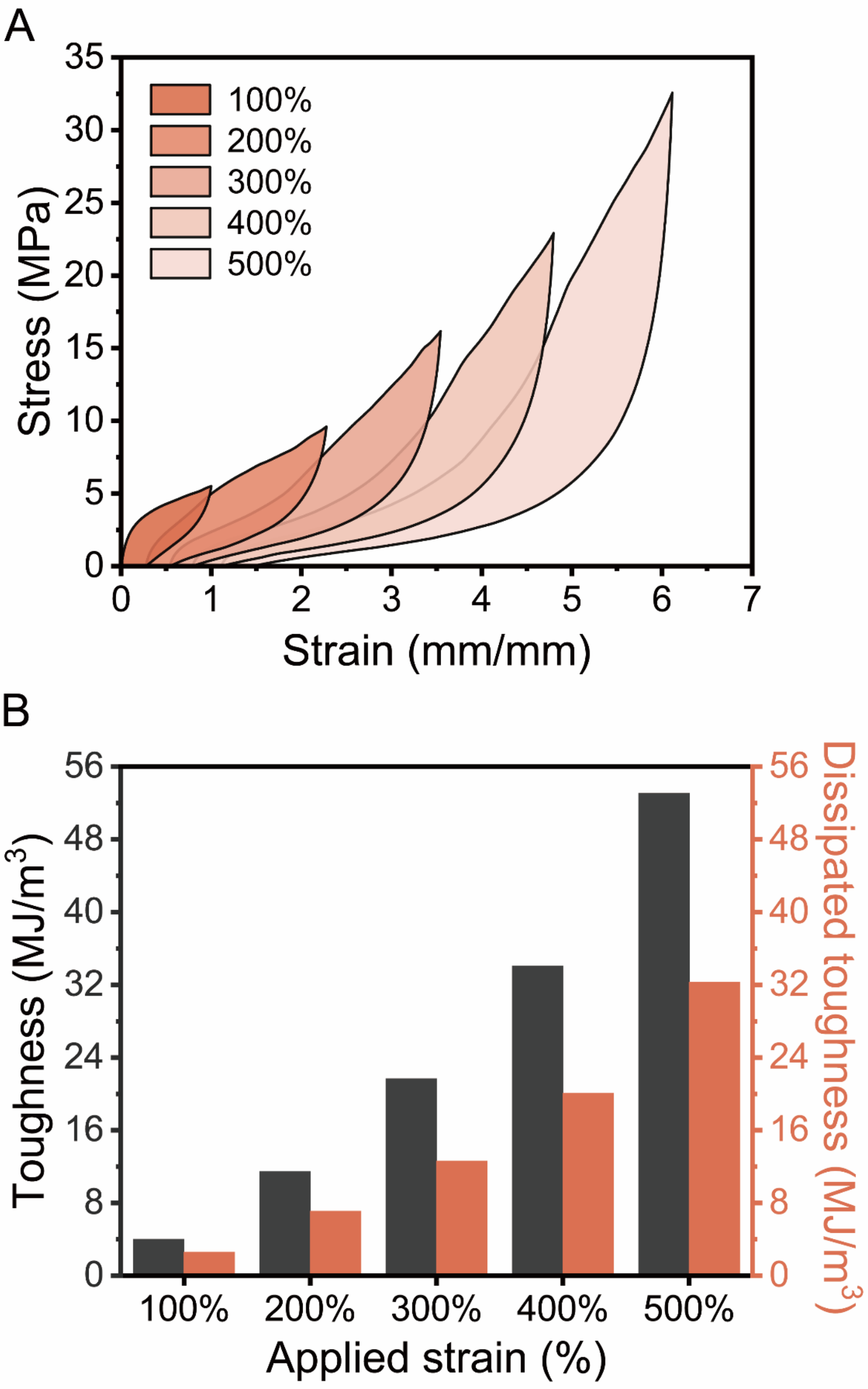

Fig. S7 **The cyclic tensile tests with gradually larger strains of DAG-PU-3** (A) Continuous cyclic tensile curves under different maximum strains. (B) The total and dissipated toughness calculated for DAG-PU-3 within five cyclic stress-strain tests in the maximum strain range from 100% to 500%.

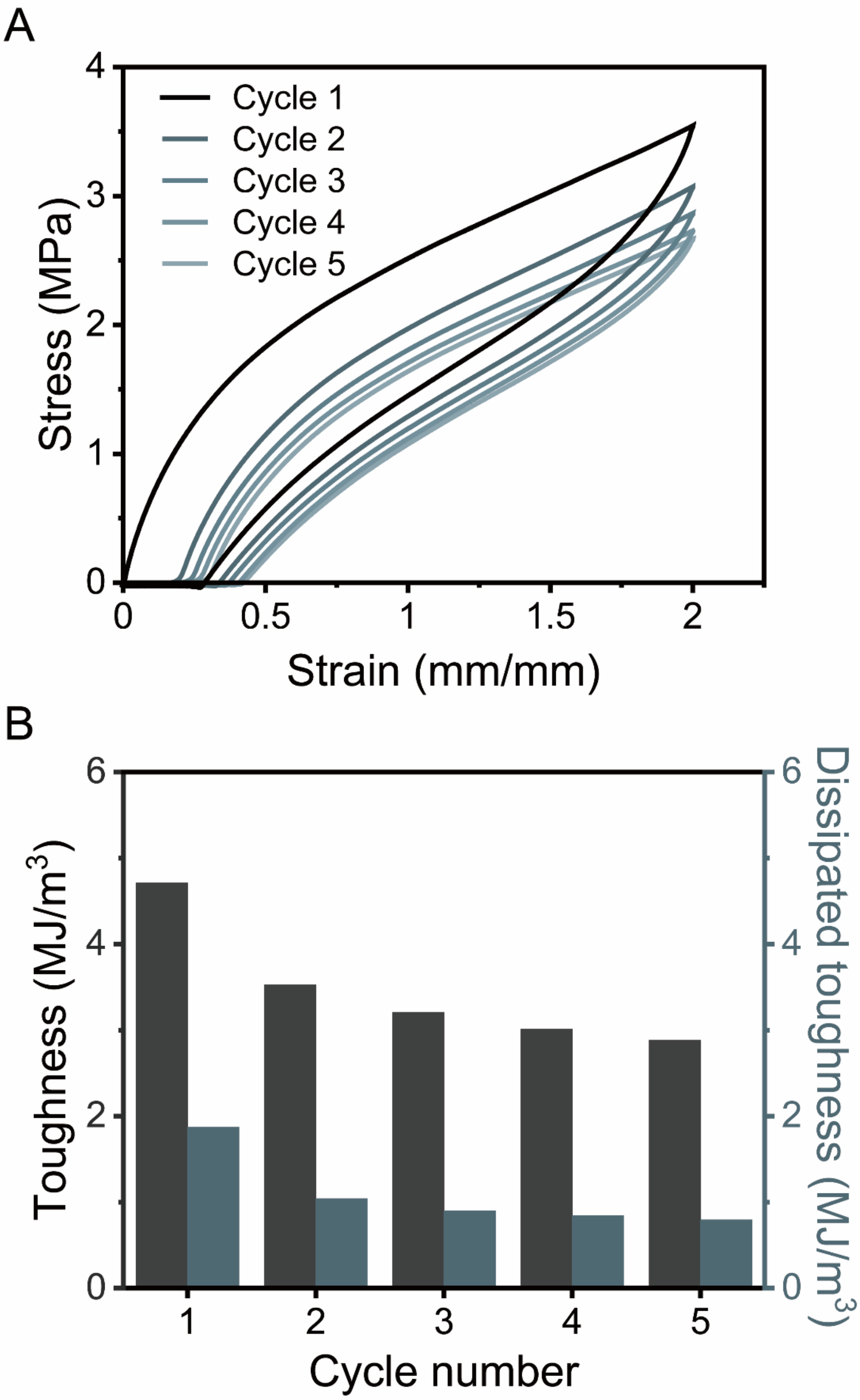

Fig. S8 **The cyclic tensile tests of DAG-PU-1** (A) Cyclic tensile test curve of the DAG-PU-1 in successive loading-unloading cycles for 5 cycles. (B) The total and dissipated toughness calculated for DAG-PU-1 within five successive loading-unloading tensile tests.

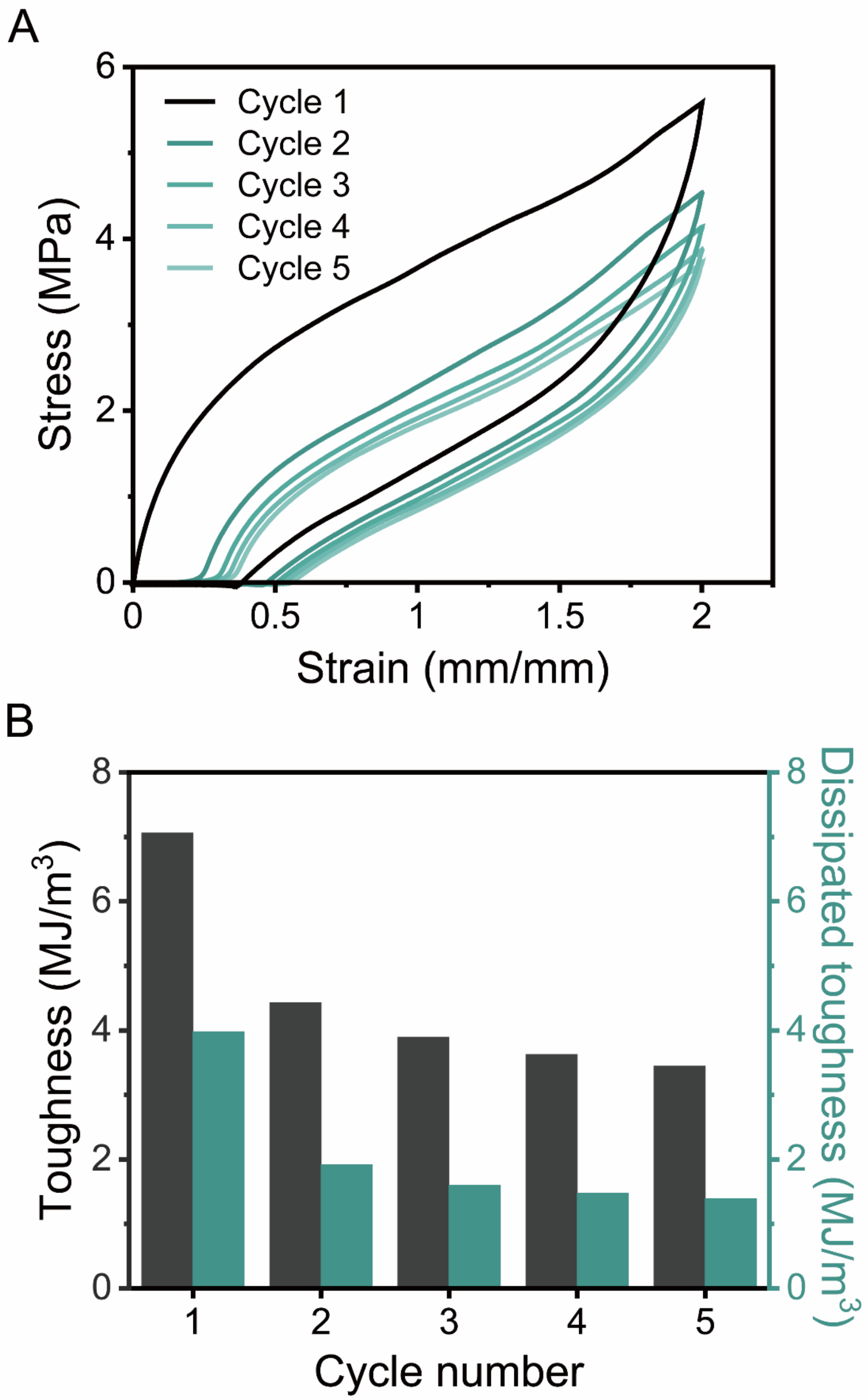

Fig. S9 **The cyclic tensile tests of DAG-PU-2** (A) Cyclic tensile test curve of the DAG-PU-2 in successive loading-unloading cycles for 5 cycles. (B) The total and dissipated toughness calculated for DAG-PU-2 within five successive loading-unloading tensile tests.

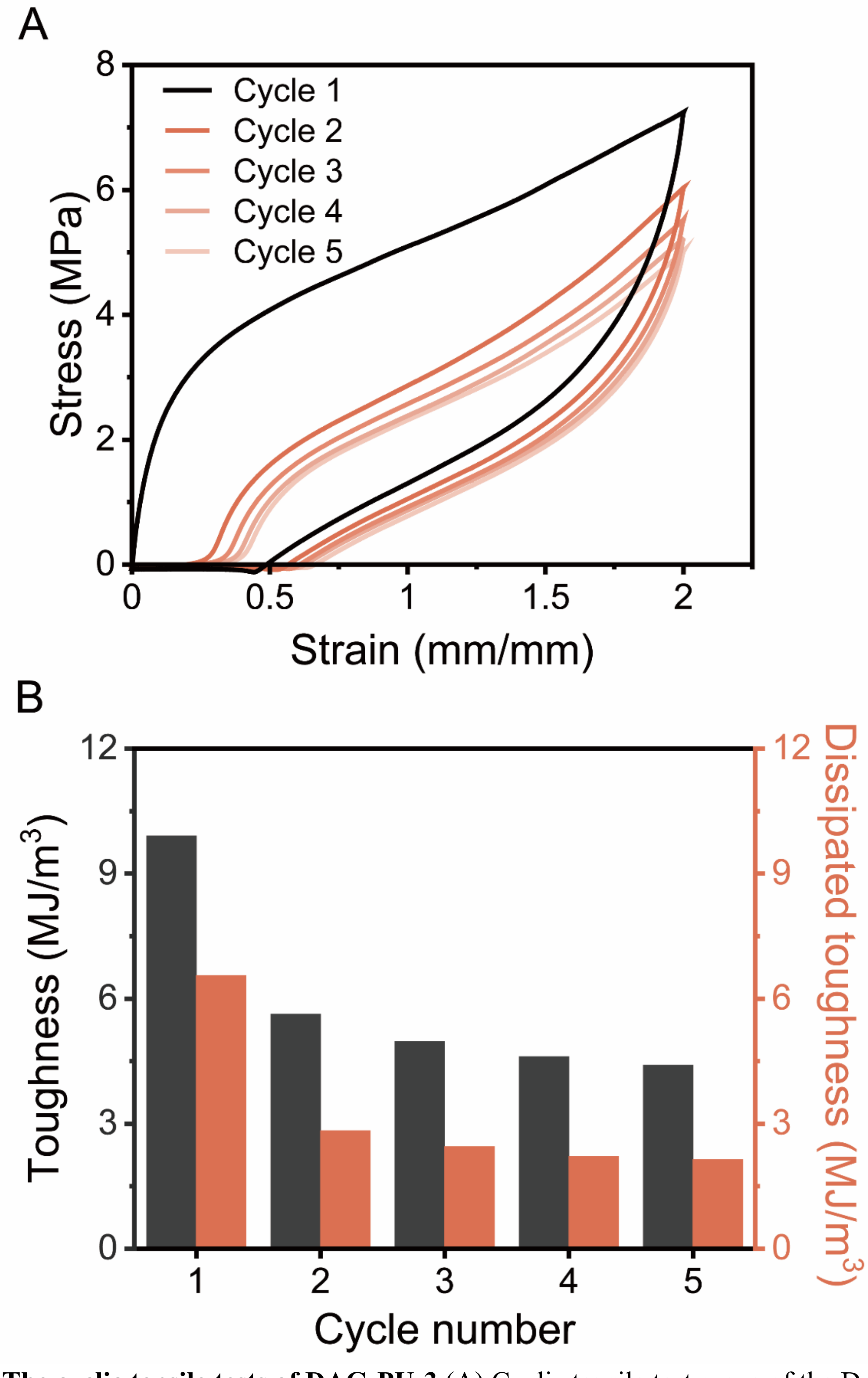

Fig. S10 **The cyclic tensile tests of DAG-PU-3** (A) Cyclic tensile test curve of the DAG-PU-3 in successive loading-unloading cycles for 5 cycles. (B) The total and dissipated toughness calculated for DAG-PU-3 within five successive loading-unloading tensile tests.

5. Thermal properties of DAG-PU-1, DAG-PU-2 and DAG-PU-3

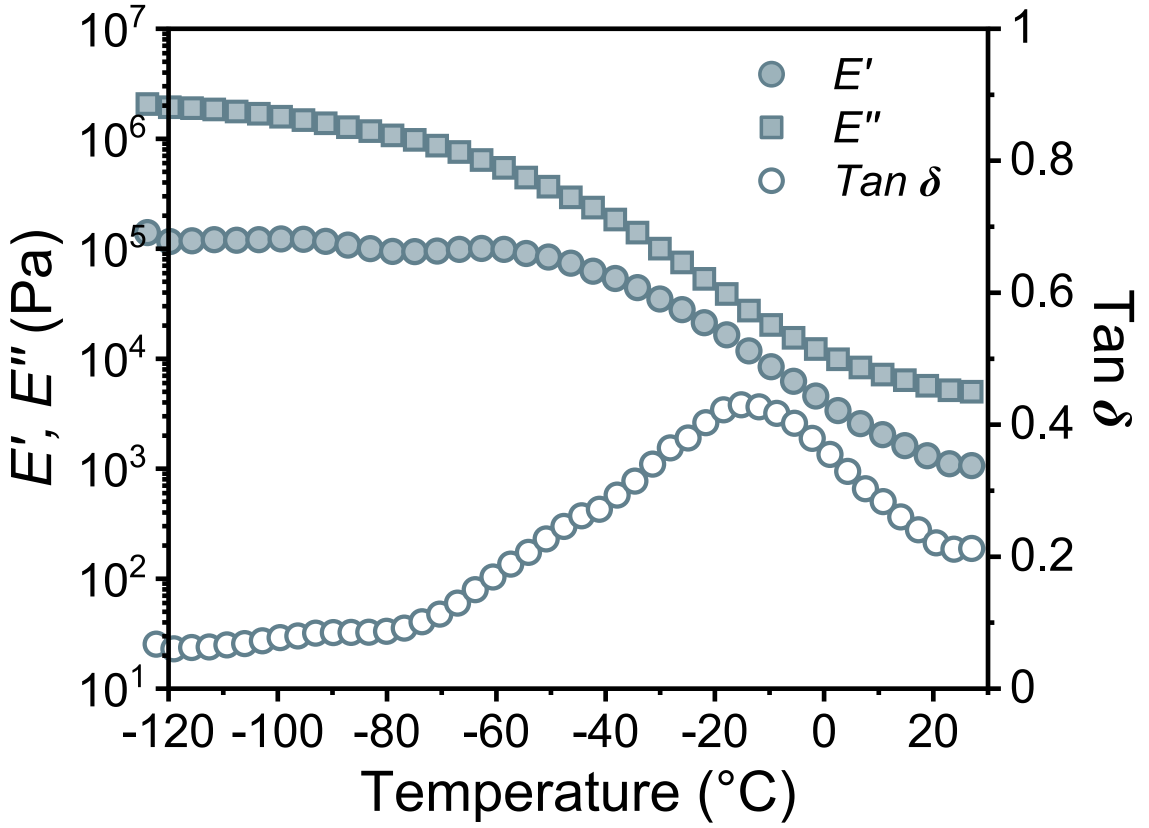

Fig. S11 Dynamic mechanical analysis (DMA) temperature sweeps of DAG-PU-1 from -120 to 25 °C.

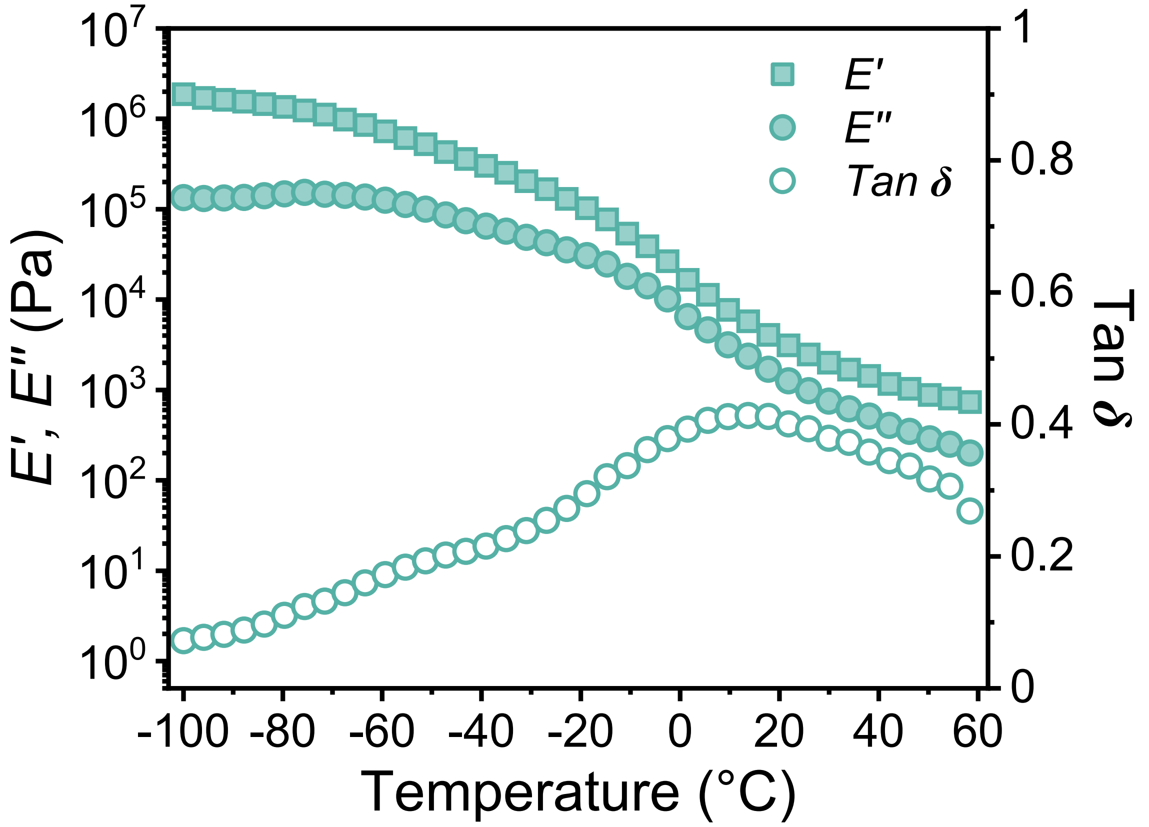

Fig. S12 Dynamic mechanical analysis (DMA) temperature sweeps of DAG-PU-2 from -100 to 60 °C

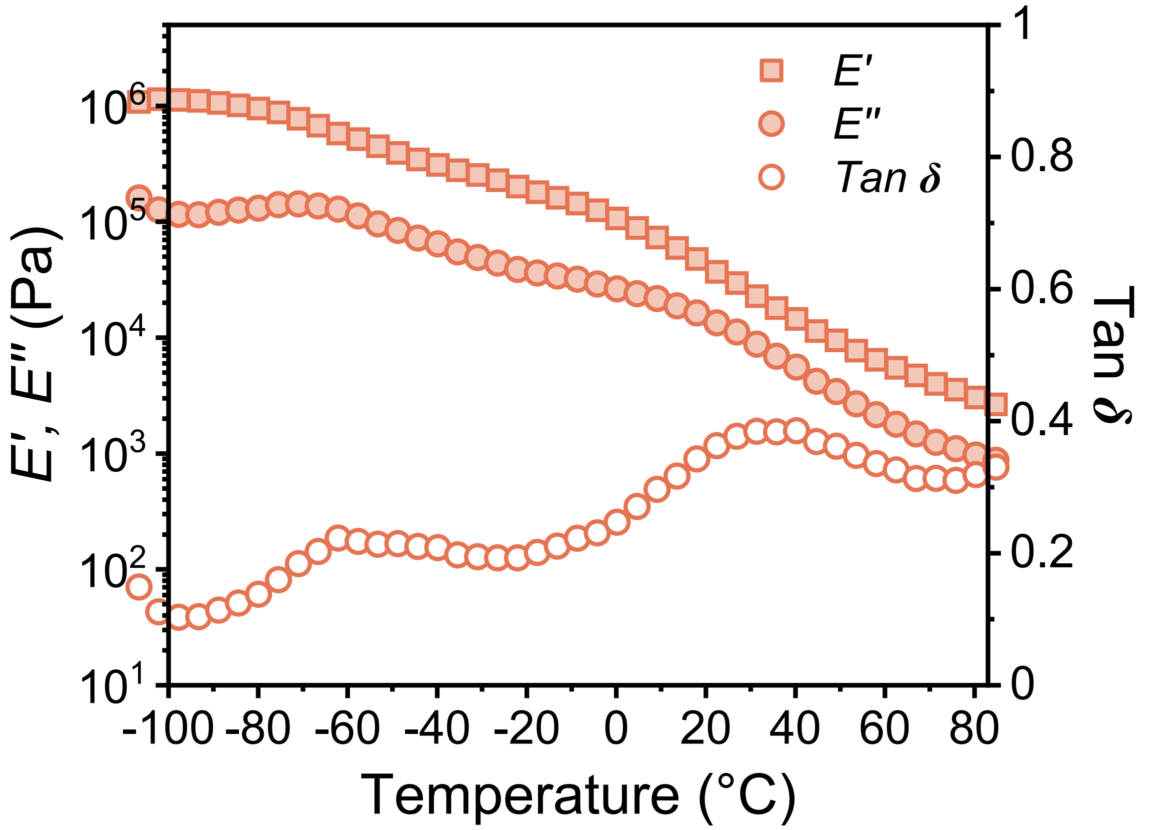

Fig. S13 Dynamic mechanical analysis (DMA) temperature sweeps of DAG-PU-3 from -100 to 80 °C

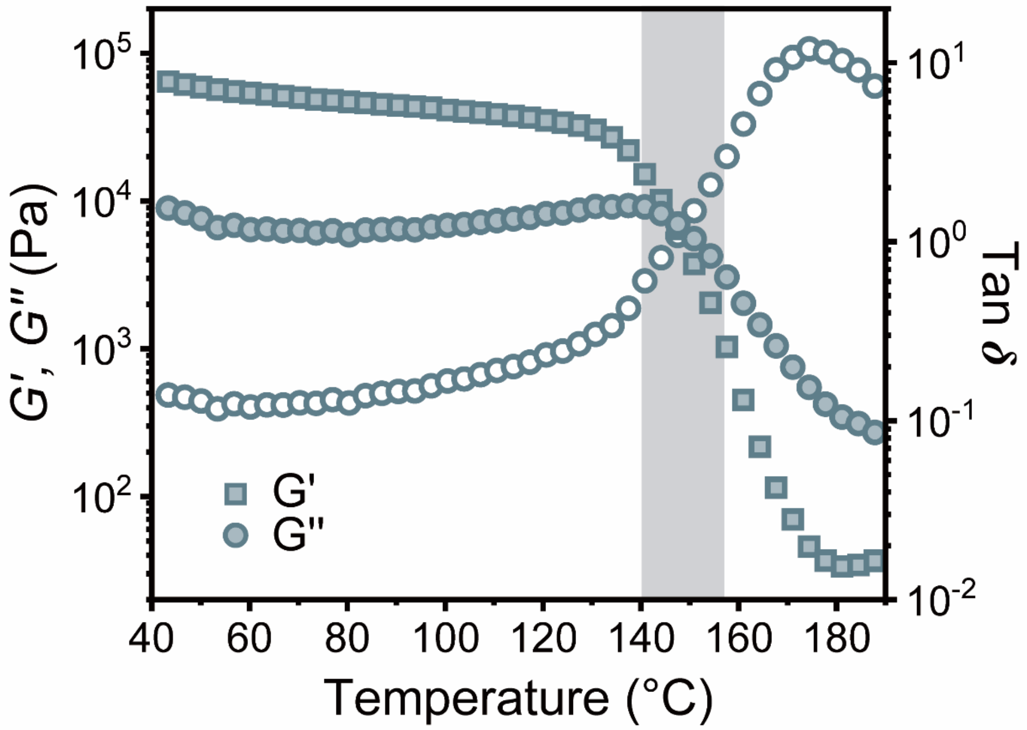

Fig. S14 Temperature sweep rheological analysis of DAG-PU-1 in the range of 40-180 °C with a heating rate of 5.0 °C/min.

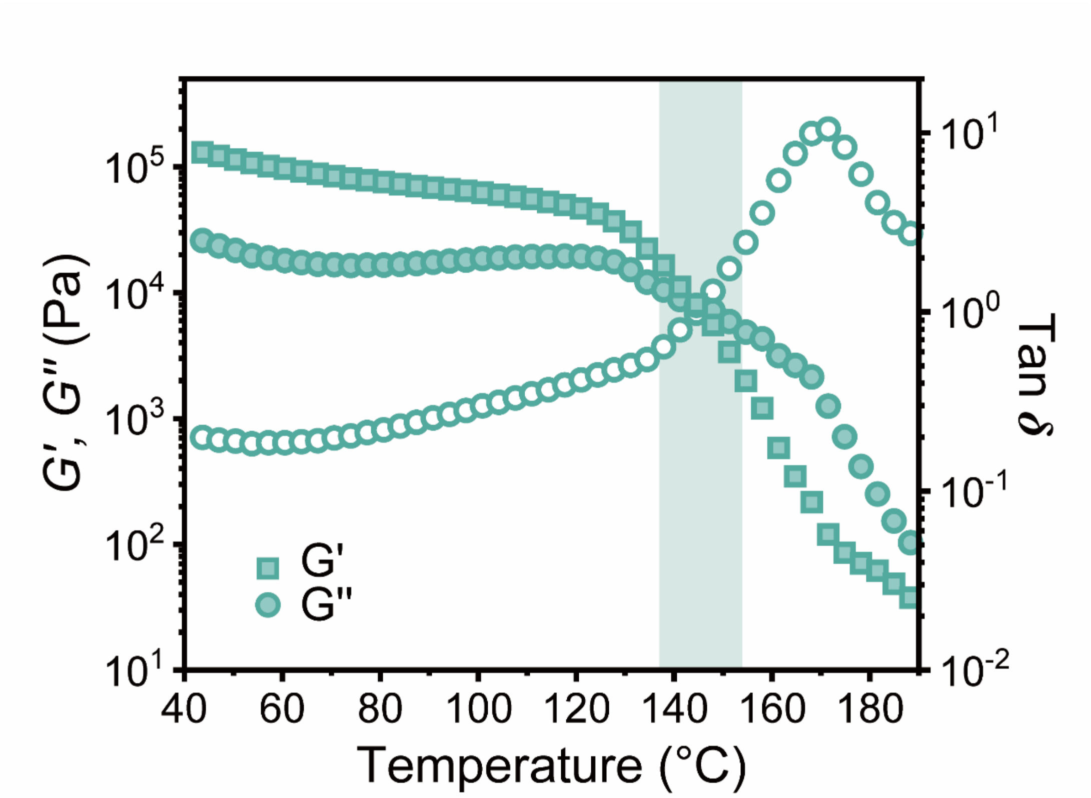

Fig. S15 Temperature sweep rheological analysis of DAG-PU-2 in the range of 40-180 °C with a heating rate of 5.0 °C/min.

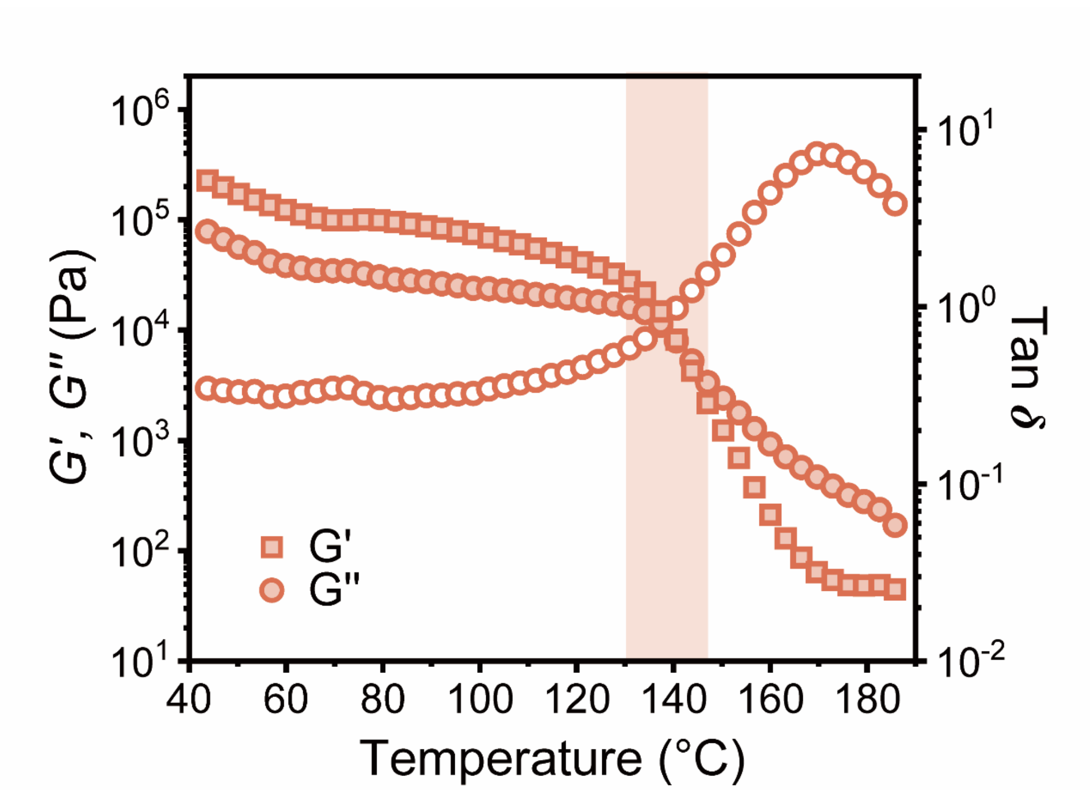

Fig. S16 Temperature sweep rheological analysis of DAG-PU-3 in the range of 40-180 °C with a heating rate of 5.0 °C/min.

Table S2 Melt mass-flow rate of DAG-PU

No./ Sample	DAG-PU-1	DAG-PU-2	DAG-PU-3
1	0.216	0.328	0.446
2	0.204	0.313	0.467
3	0.208	0.318	0.425
4	0.203	0.318	0.414
5	0.210	0.331	0.398
Average value (g/1 min)	0.208	0.322	0.43
Average value (g/10 min)	12.5	19.3	26.3

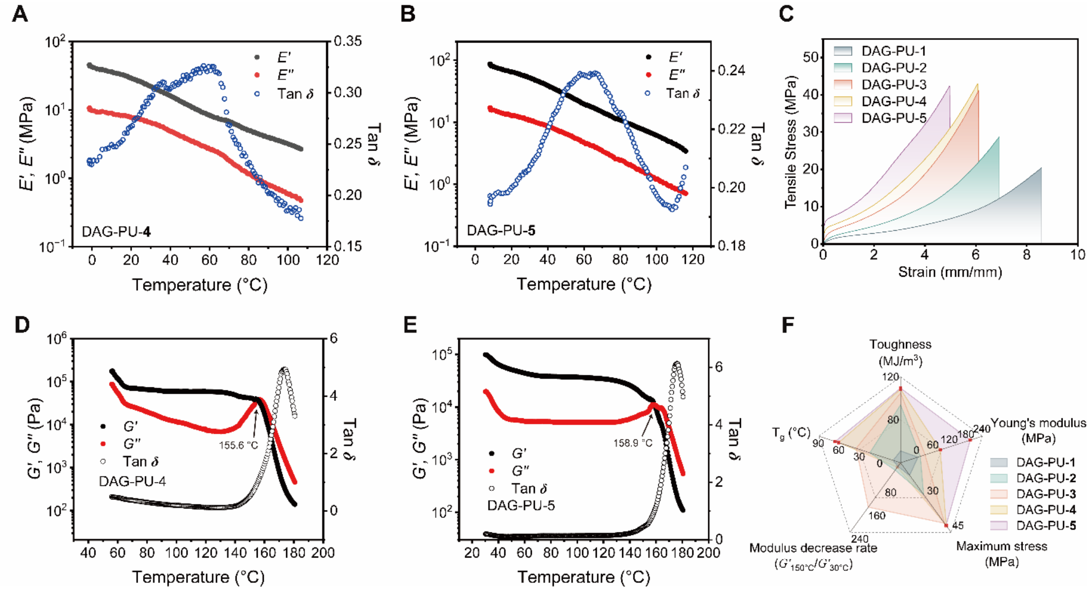

Fig. S17 **Mechanical and processing performance of DAG-PUs** (A-B) DMA temperature sweeps of (A) DAG-PU-4 and (B) DAG-PU-5. (C) Stress-strain curves of DAG-PUs. (D-E) Temperature sweep rheological analysis of (D) DAG-PU-4 and (E) DAG-PU-5. (F) Comparison of the mechanical properties and processability of DAG-PUs.

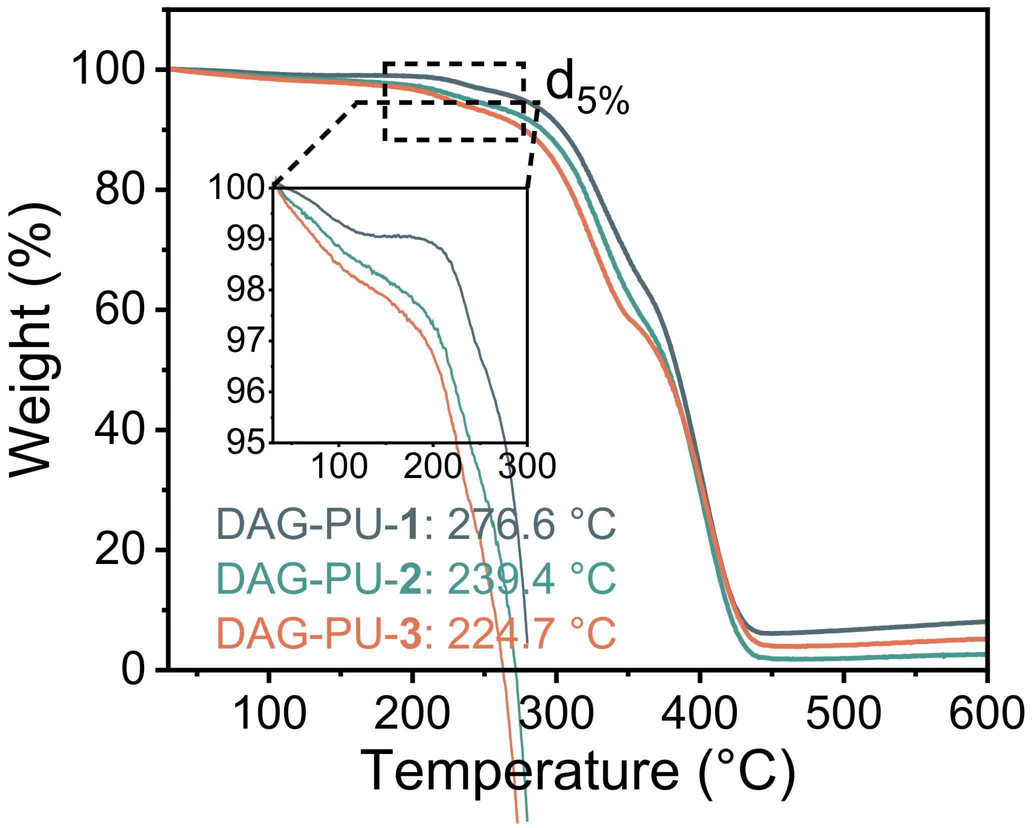

Fig. S18 TGA curves of DAG-PU-1, DAG-PU-2, and DAG-PU-3 recorded under N_2 flow (50 mL/min) with a heating rate of 20 °C/min

6. Self-healing and processability of DAG-PU-1, DAG-PU-2 and DAG-PU-3

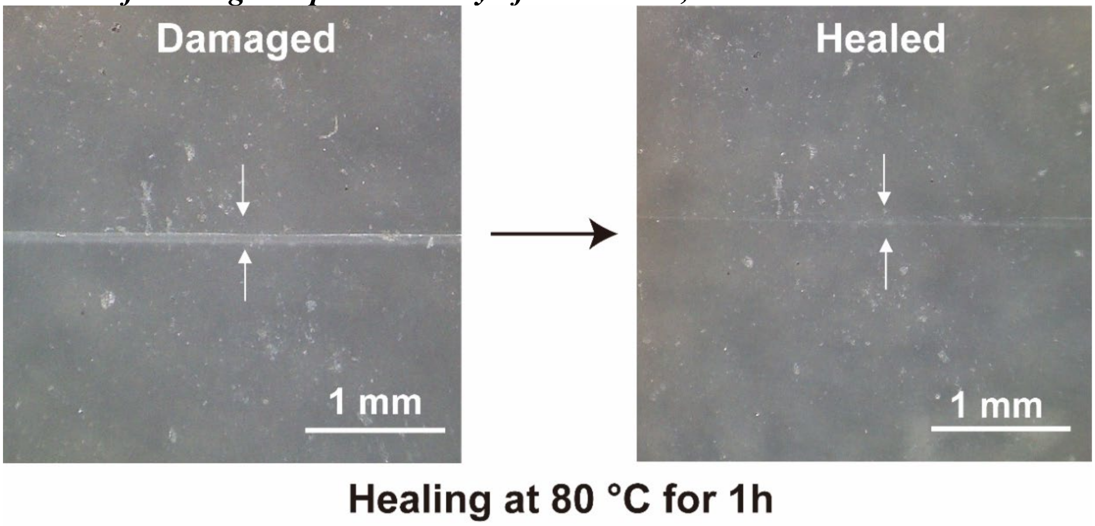

Fig. S19 Optical microscopy images of DAG-PU-3 film after being scratched. It showed a rapid self-healing with an almost complete recovery within 1 h at 80 °C.

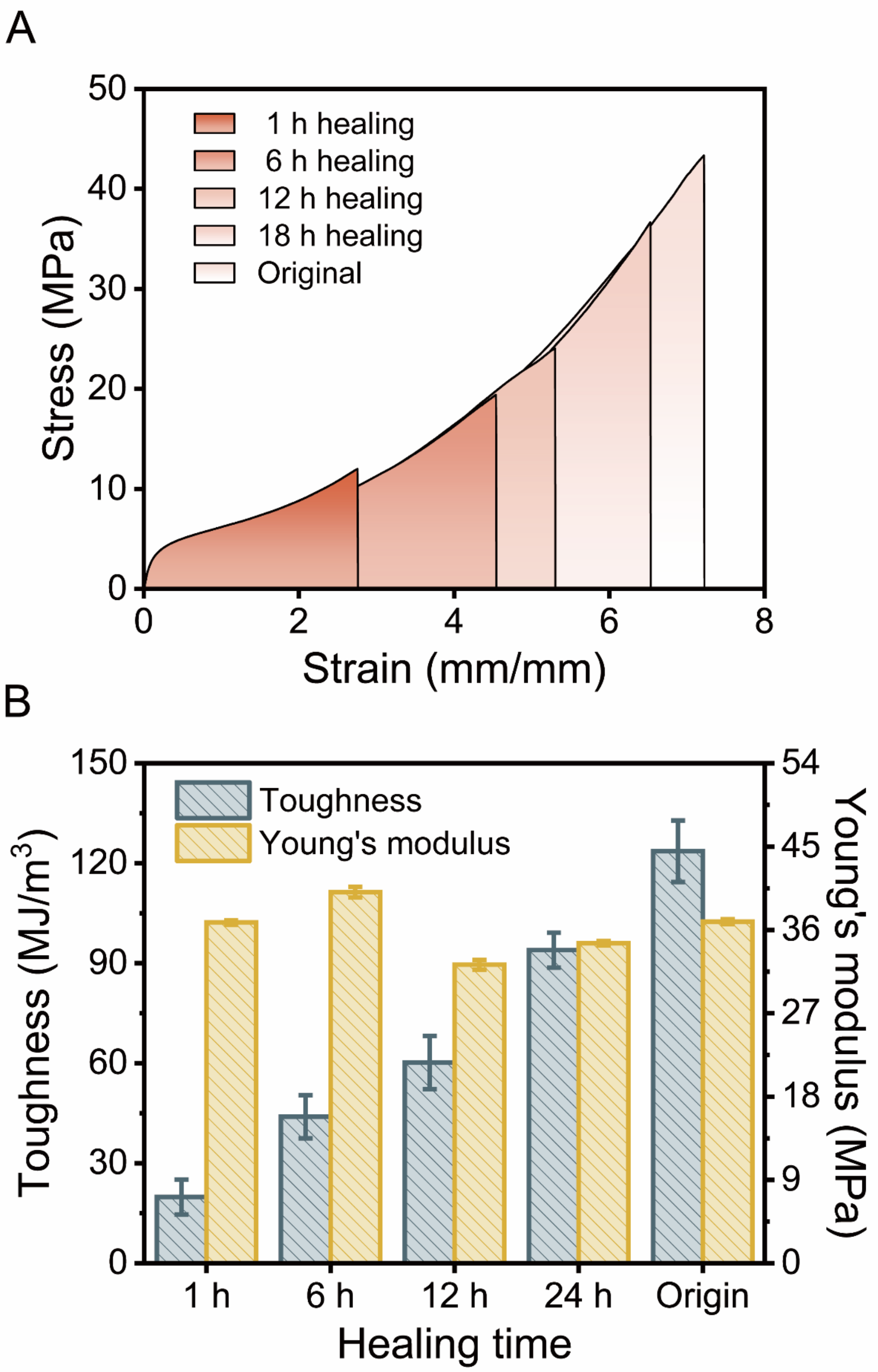

Fig. S20 **The self-healing properties of DAG-PU-3** (A) Stress-strain curves of the initial and healed DAG-PU-3 specimens after healing for 3, 6, 12, and 24 h at 80 °C, respectively. (B) Toughness and Young's modulus of DAG-PU-3 were calculated based on the stress-strain curves of the initial and healed specimens.

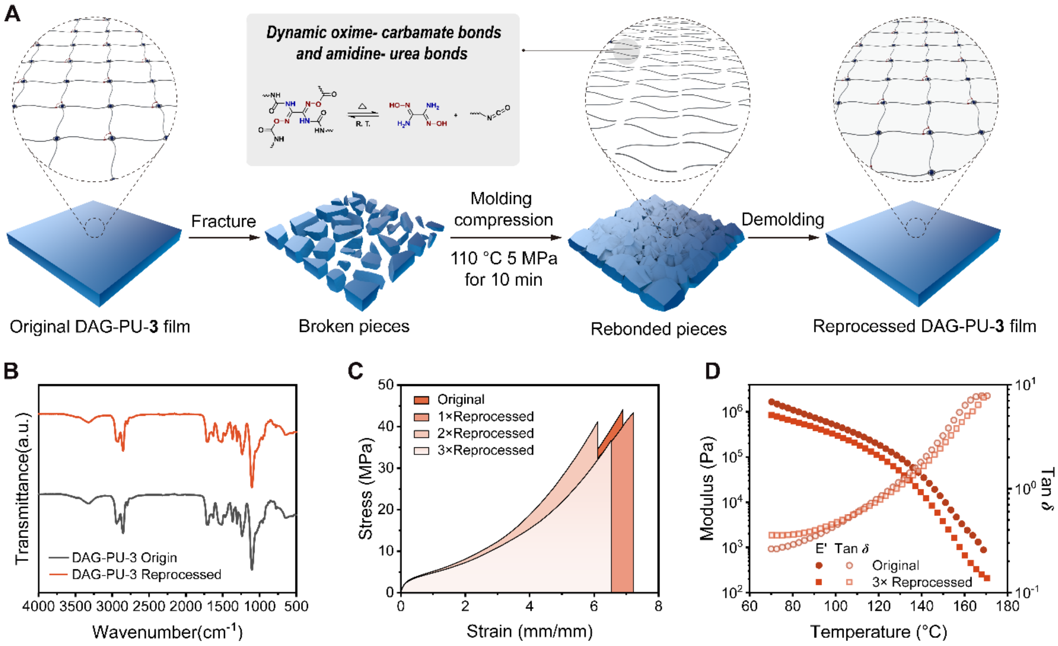

Fig. S21 **The reprocessing performance of DAG-PU-3** (A) Schematic illustration of the reprocessing process of DAG-PU as well as the mechanism of dynamic oxime-carbamate bonds and amidine-urea bonds. (B) FTIR spectra, (C) stress−strain curves and (D) temperature sweep rheological analysis of virgin and reprocessed DAG-PU-3 specimens, suggestive of the retention of chemical and mechanical integrity.

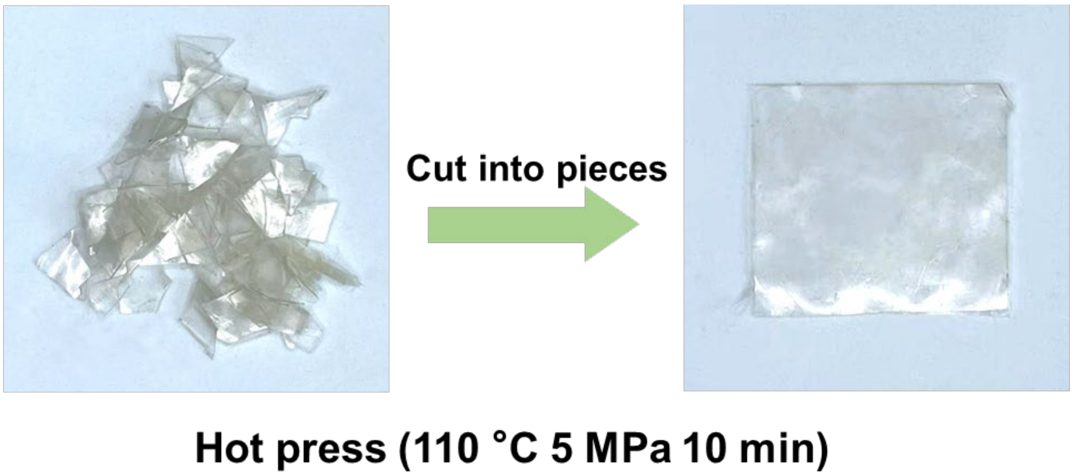

Fig. S22 Photographs of the reprocessed samples by hot pressing at 110 °C with a pressure of 5 MPa for 10 min.

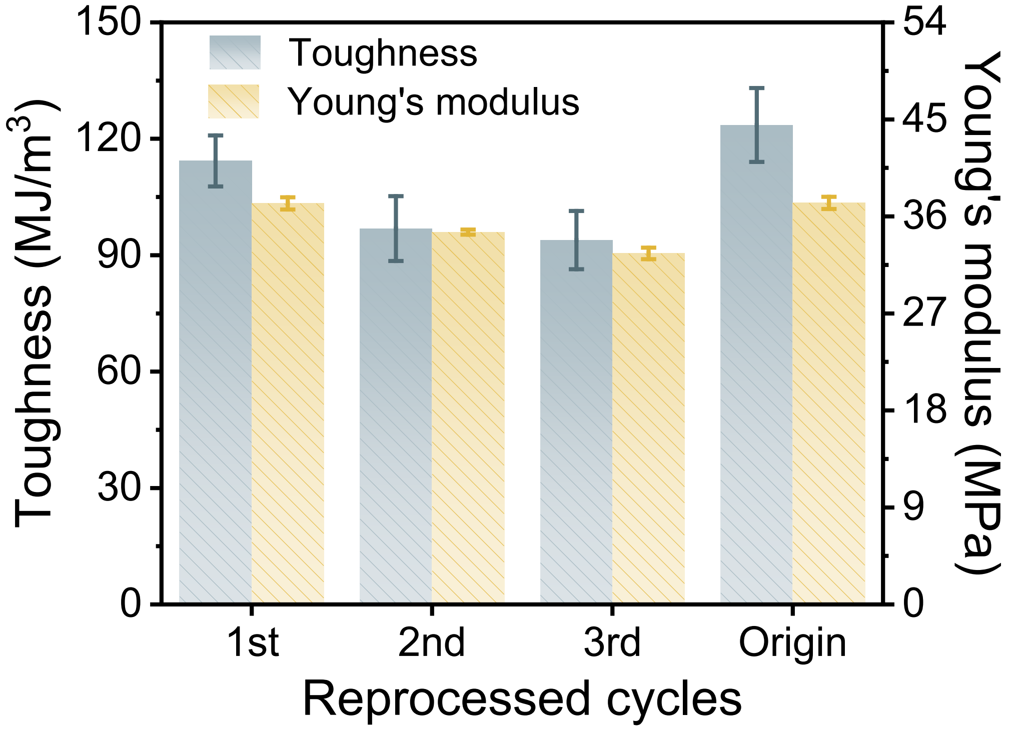

Fig. S23 Toughness and Young's modulus of DAG-PU-3 calculated based on the stress-strain curves of the initial and reprocessed specimens

7. *Syntheses of small-molecule model*

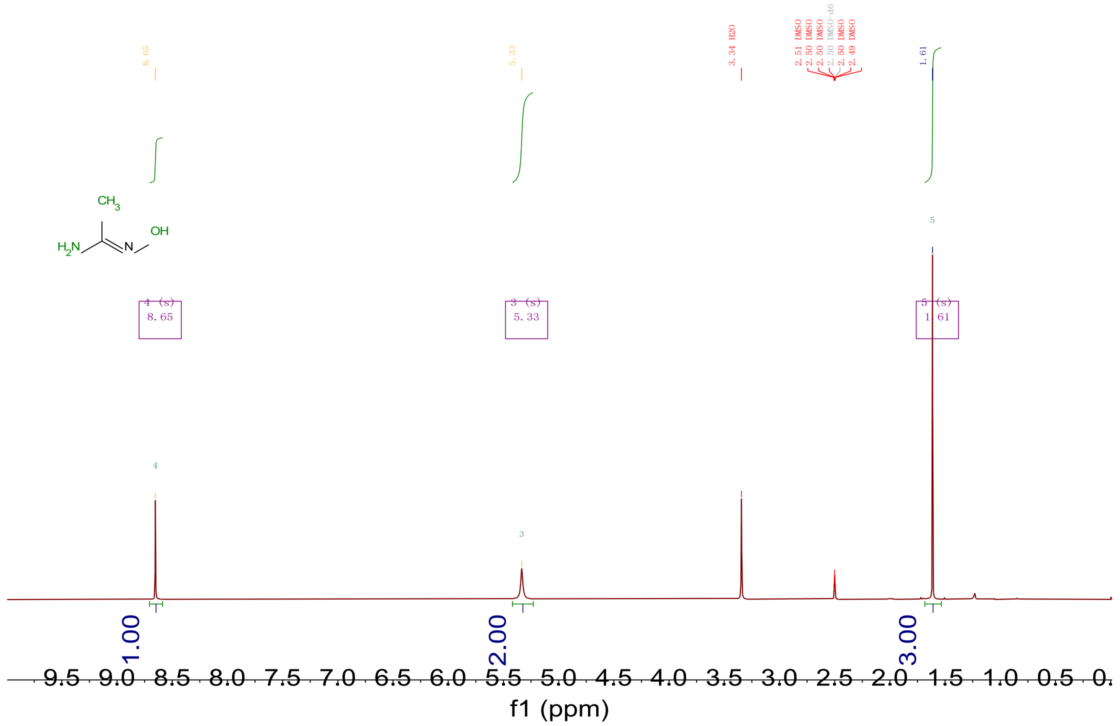

Fig. S24 ^1H NMR spectrum of compound **c** (n-hydroxyacetamidine). ^1H NMR (600 MHz, DMSO-*d6*) δ 8.65 (s, 1H), 5.33 (s, 2H), 1.61 (s, 3H).

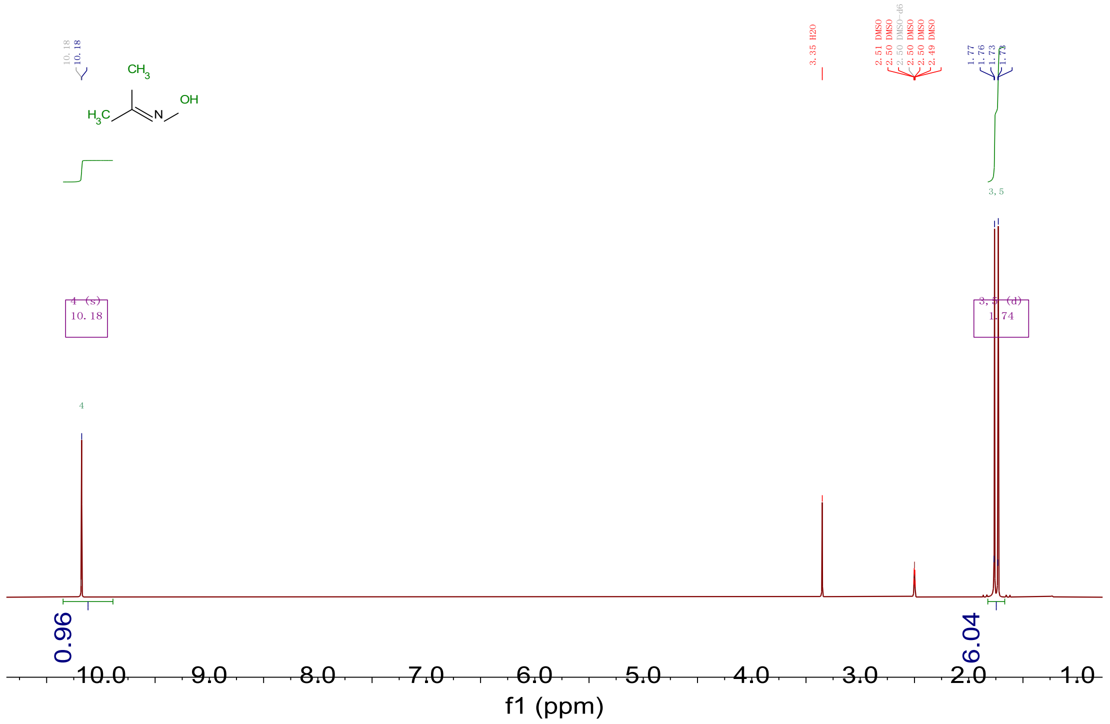

Fig. S25 ^1H NMR spectrum of compound **d** (acetoxime). ^1H NMR (600 MHz, DMSO-*d*6) δ 10.18 (s, 1H), 1.74 (d, J = 20.5 Hz, 6H).

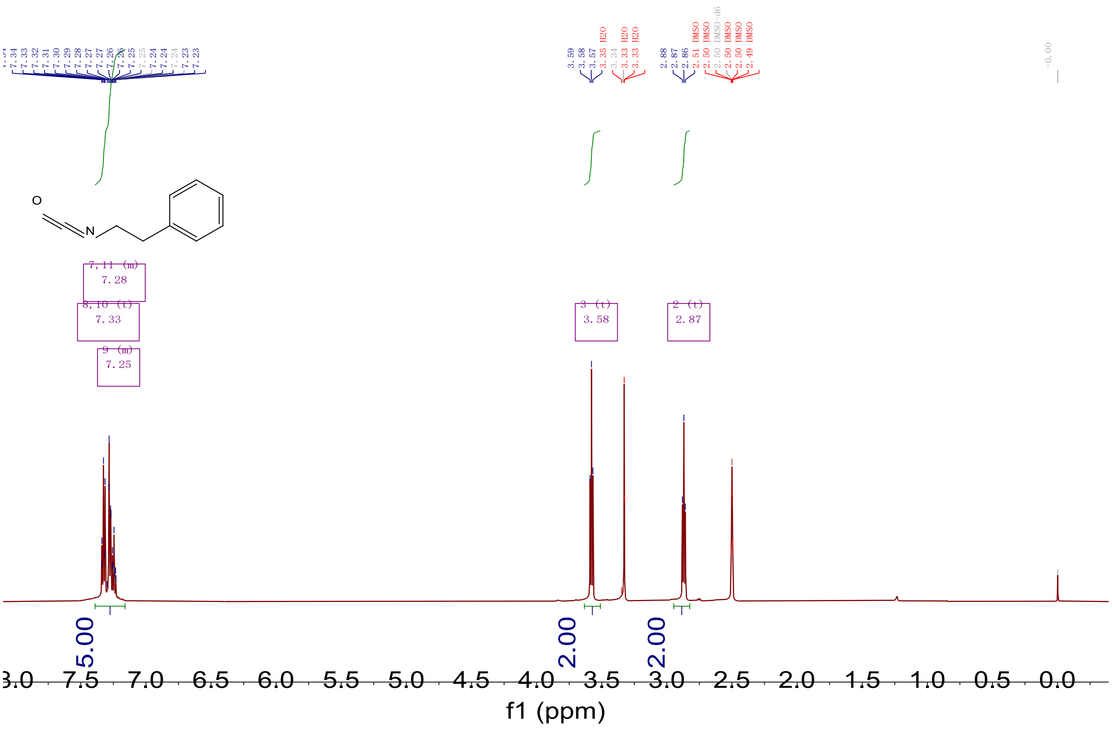

Fig. S26 ^{1}H NMR spectrum of compound **b** (phenethyl isocyanate). ^{1}H NMR (600 MHz, DMSO-*d6*) δ 7.33 (t, $J = 7.6$ Hz, 2H), 7.30 – 7.25 (m, 2H), 7.27 – 7.22 (m, 1H), 3.58 (t, $J = 6.7$ Hz, 2H), 2.87 (t, $J = 6.7$ Hz, 2H)

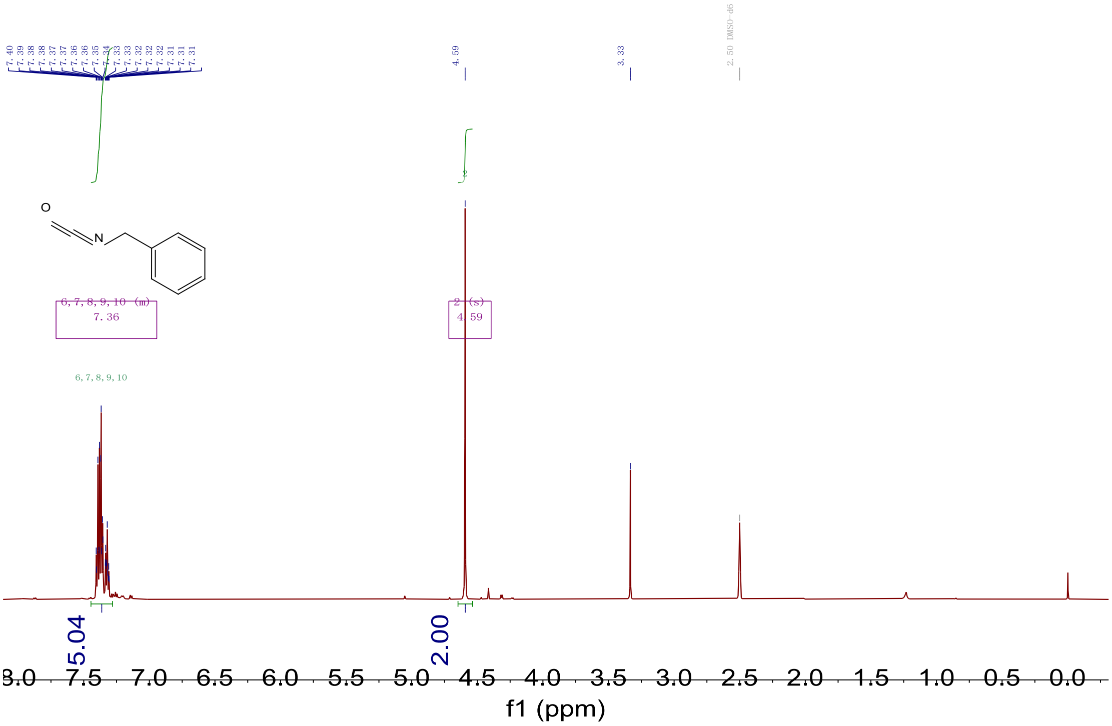

Fig. S27 ^{1}H NMR spectrum of compound **a** (benzyl isocyanate). ^{1}H NMR (600 MHz, DMSO-*d6*) δ 7.44 – 7.28 (m, 5H), 4.59 (s, 2H).

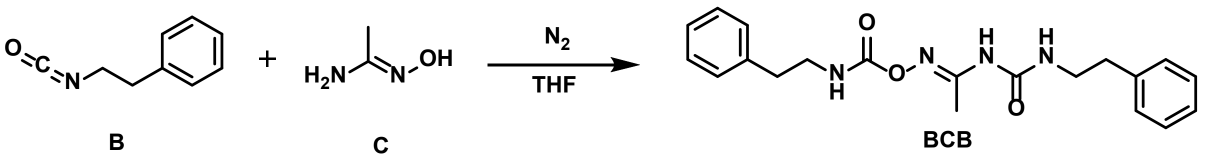

Fig. S28 Synthesis of small-molecule model compound **bcb**

N-hydroxyacetamidine (compound **c**, 0.74 g, 10 mmol) was dissolved in THF (5 mL). Phenethyl isocyanate (compound **b**, 2.96 g, 20 mmol) was added to react at 30 °C under a nitrogen atmosphere for 6 h. The solution was then added dropwise to n-hexane to generate a white precipitate at -20 °C. The white precipitate was dried at room temperature under vacuum and compound a was obtained.

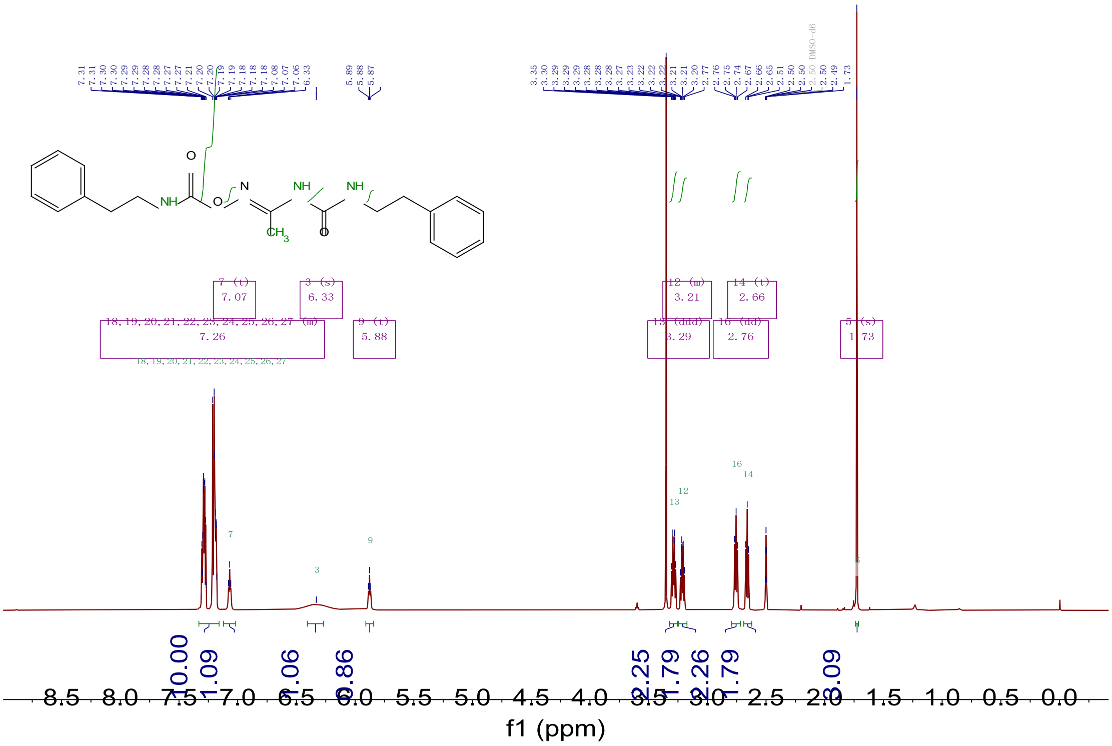

Fig. S29 ^{1}H NMR spectrum of compound **bcb**

^{1}H NMR (600 MHz, DMSO-*d6*) δ 7.33 – 7.16 (m, 9H), 7.07 (t, *J* = 5.9 Hz, 1H), 6.33 (s, 2H), 5.88 (t, *J* = 5.8 Hz, 1H), 3.29 (ddd, *J* = 8.7, 7.5, 6.0 Hz, 2H), 3.25 – 3.17 (m, 2H), 2.76 (dd, *J* = 8.5, 6.6 Hz, 2H), 2.66 (t, *J* = 7.3 Hz, 2H), 1.73 (s, 3H).

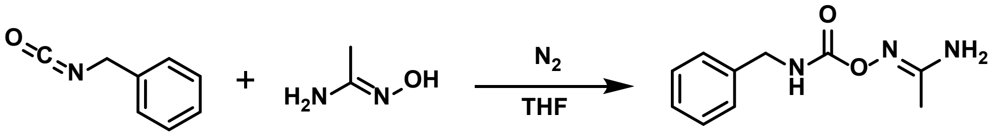

A C AC

Fig. S30 Synthesis of small-molecule model compound **ac**

N-hydroxyacetamidine (compound **c**, 0.74 g, 10 mmol) was dissolved in THF (5 mL). benzyl isocyanate (compound **a**, 1.33 g, 10 mmol) was added to react at 30 °C under a nitrogen atmosphere for 6 h. The solution was then added dropwise to n-hexane to generate a white precipitate at -20 °C. The white precipitate was dried at room temperature under vacuum and compound a was obtained.

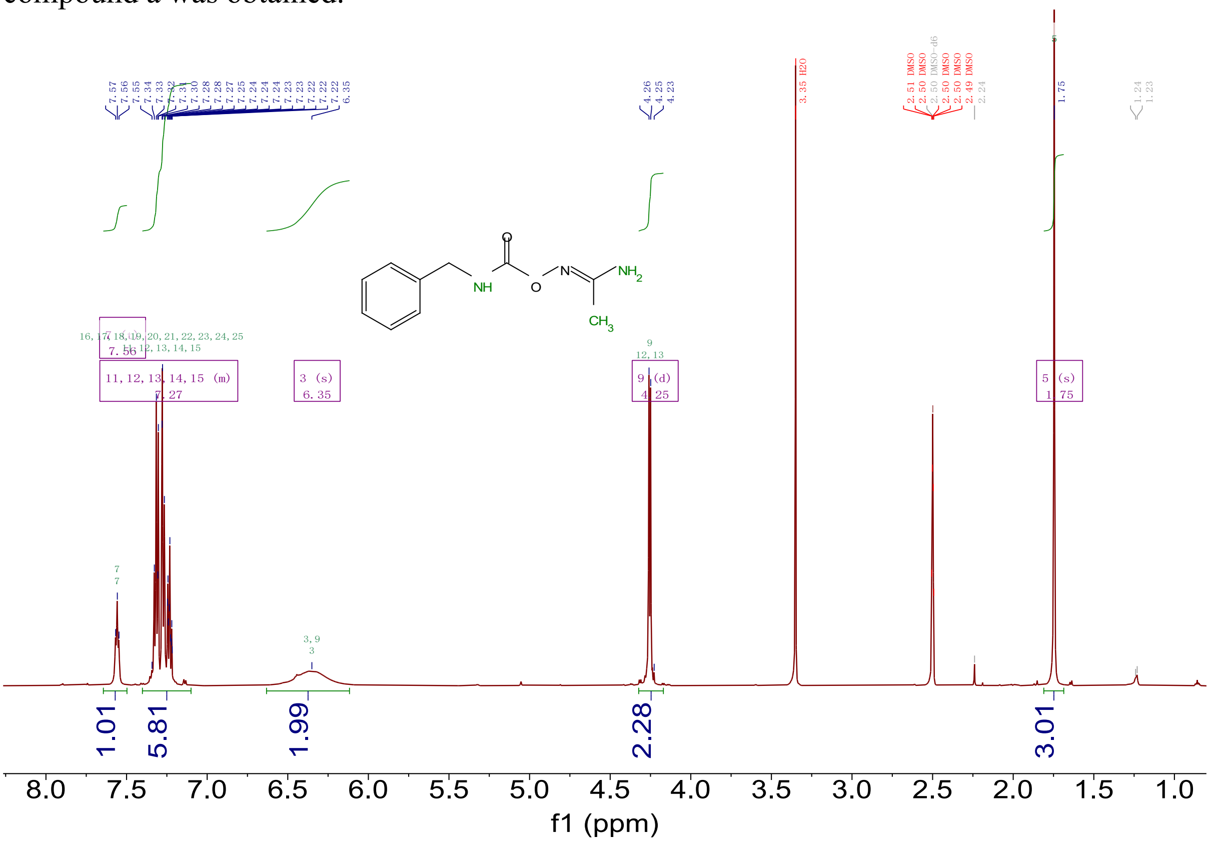

Fig. S31 ^{1}H NMR spectrum of compound **ca**

^{1}H NMR (600 MHz, DMSO-d_6) δ 7.56 (t, J = 6.2 Hz, 1H), 7.40 – 7.19 (m, 5H), 6.35 (s, 2H), 4.25 (d, J = 6.3 Hz, 2H), 1.75 (s, 3H).

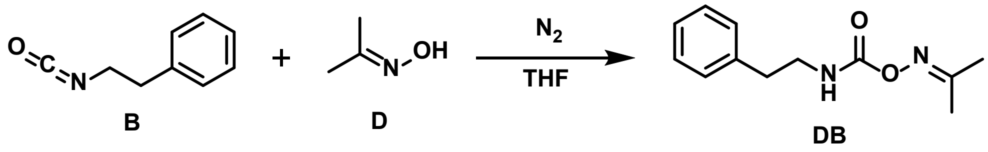

Fig. S32 Synthesis of small-molecule model compound **db**

Acetoxime (compound **d**, 0.73 g, 10 mmol) was dissolved in THF (5 mL). Phenethyl isocyanate (compound **b**, 1.47 g, 10 mmol) was added to react at 30 °C under a nitrogen atmosphere for 6 h. The solution was then added dropwise to n-hexane to generate a white precipitate at -20 °C. The white precipitate was dried at room temperature under vacuum and compound **a** was obtained.

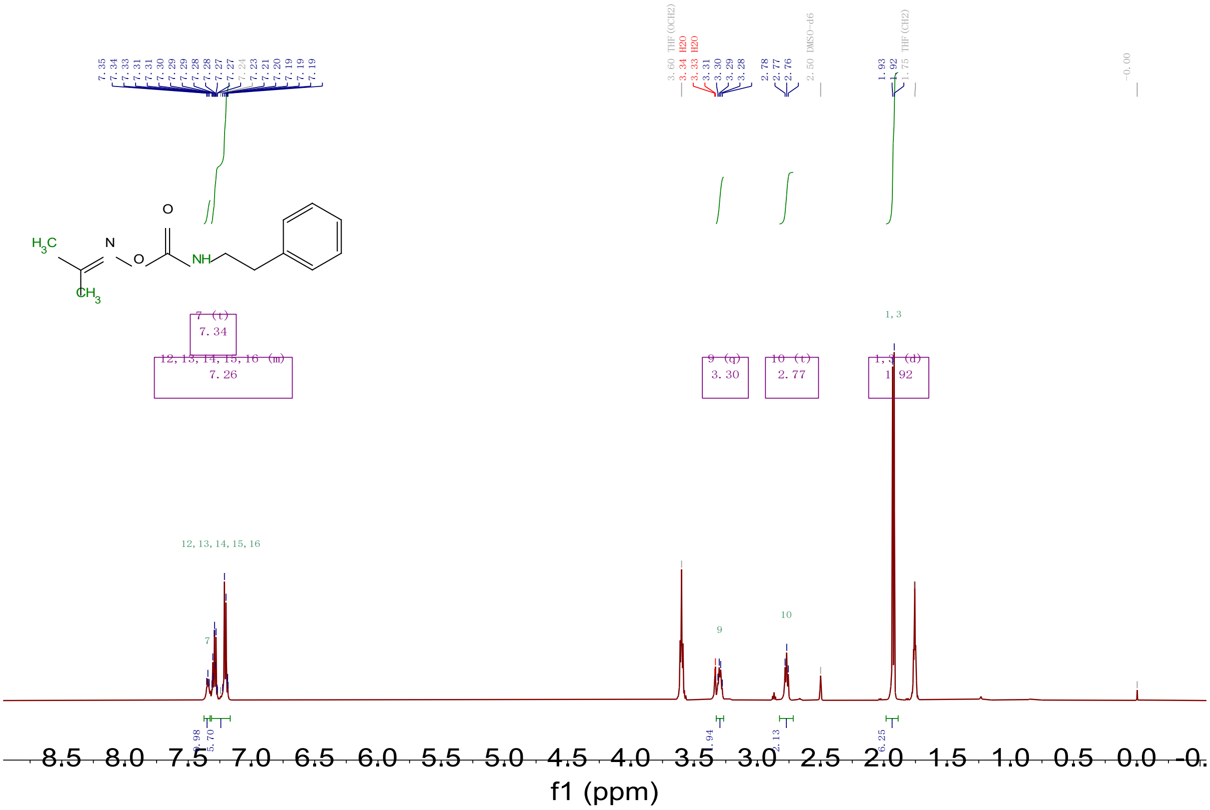

Fig. S33 ^{1}H NMR spectrum of compound **db**

^{1}H NMR (600 MHz, DMSO-*d6*) δ 7.89 (t, J = 6.2 Hz, 1H), 7.45 – 7.11 (m, 5H), 4.27 (d, J = 6.2 Hz, 2H), 1.95 (d, J = 5.3 Hz, 6H).

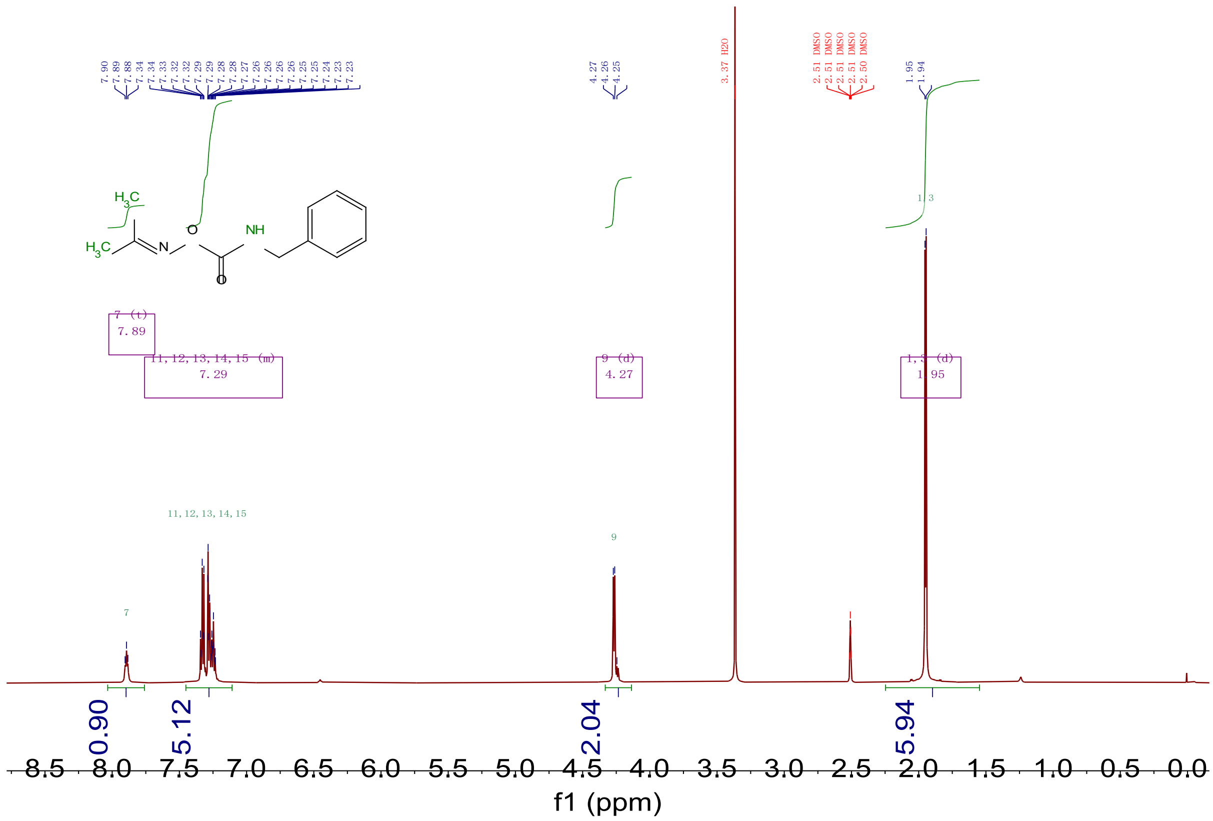

Fig. S34 ^{1}H NMR spectrum of compound **da**

^{1}H NMR (600 MHz, DMSO-*d6*) δ 7.56 (t, *J* = 6.3 Hz, 1H), 7.38 – 7.19 (m, 5H), 6.35 (s, 2H), 4.26 (d, *J* = 6.3 Hz, 2H), 1.75 (s, 3H).

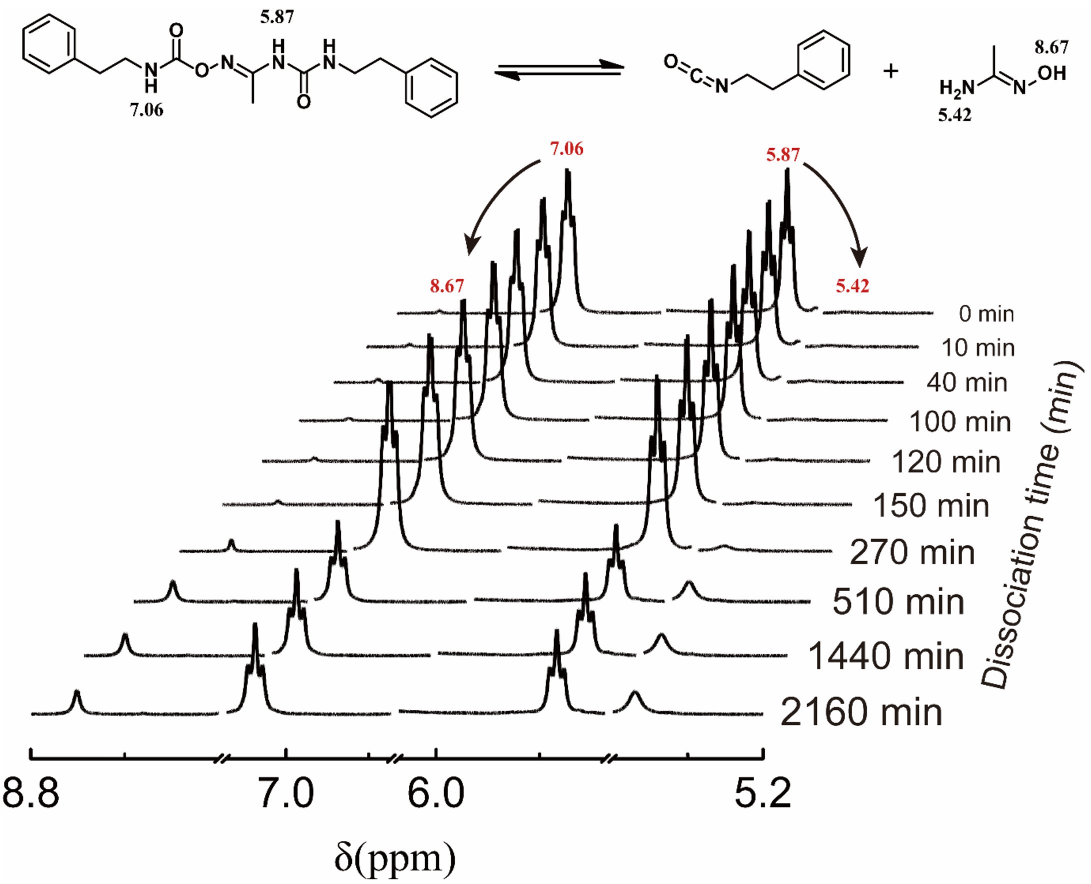

Fig. S35 *In situ* ^{1}H NMR spectra of compound **bcb**. Proton peaks at 5.42 ppm and 8.67 ppm appeared and their intensities gradually increased, indicating the generation of compound **b**.

Table S3 The Composition of Control-PU and DAG-PU-3

Sample	DAG-PU-**3**	Control-PU
PTMG-1000	4 mmol	4 mmol
IPDI	10 mmol	16 mmol
DAG	3 mmol	/
DMG	/	6 mmol
Pentaerythritol	/	3 mmol
DBTDL	0.2 wt%	0.2 wt%

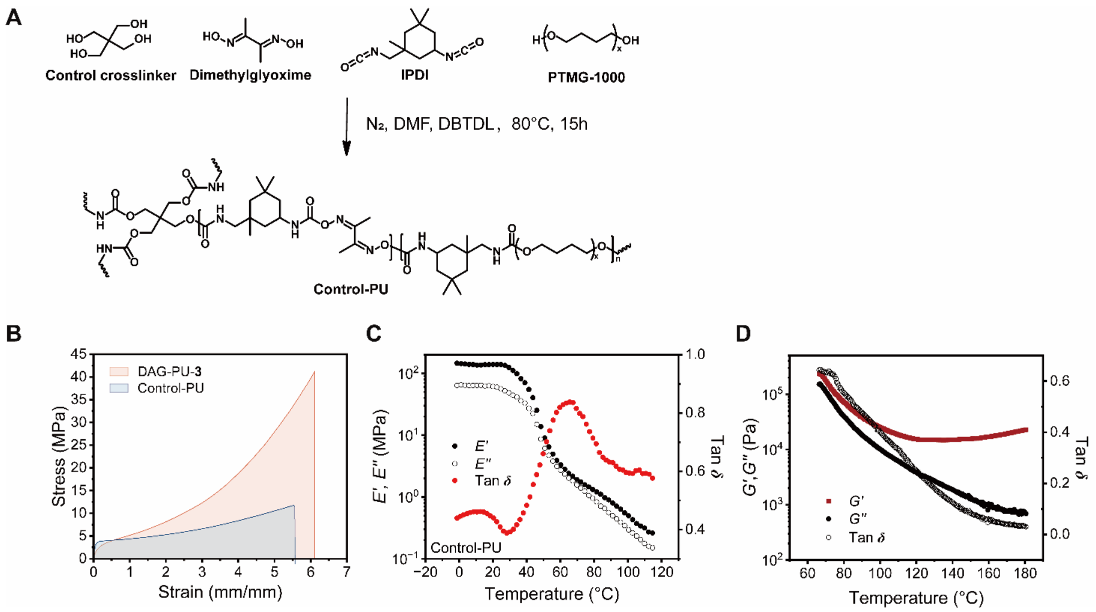

Fig. S36 **The mechanical and thermal property of control-PU** (A) Synthetic route of the control-PU; (B) The mechanical property of DAG-PU-3 and control-PU; (C) DMA temperature sweeps of control-PU; (D) Temperature sweep rheological analysis of control-PU

8. Rheological test of DAG-PU-1, DAG-PU-2 and DAG-PU-3

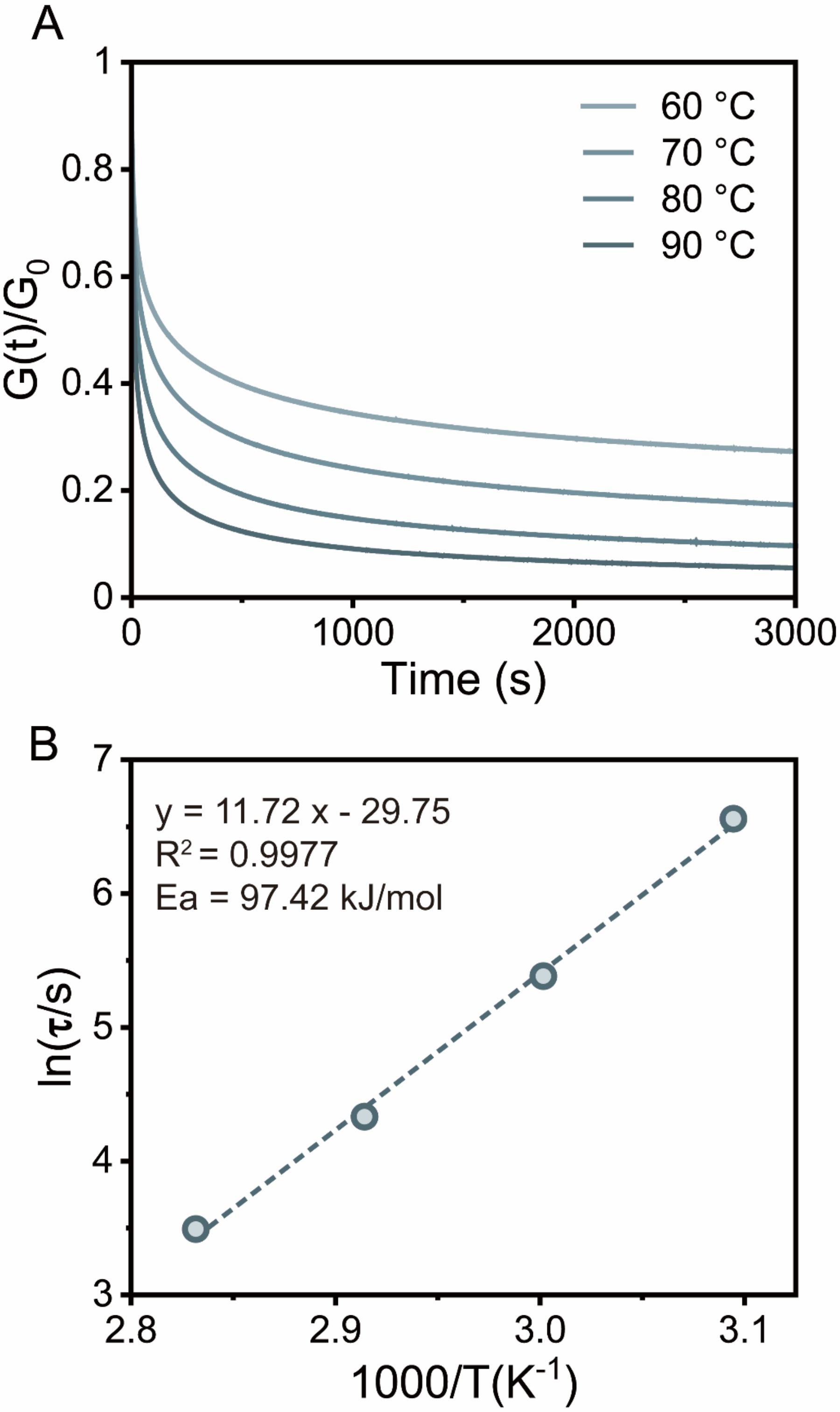

Fig. S37 **Normalized variable temperature stress relaxation of DAG-PU-1** (A) Stress relaxation curves at 60 °C, 70 °C, 80 °C and 90 °C of DAG-PU-1. (B) Plots of ln (τ*) -1000/T and linear fit and E_a for DAG-PU-1.

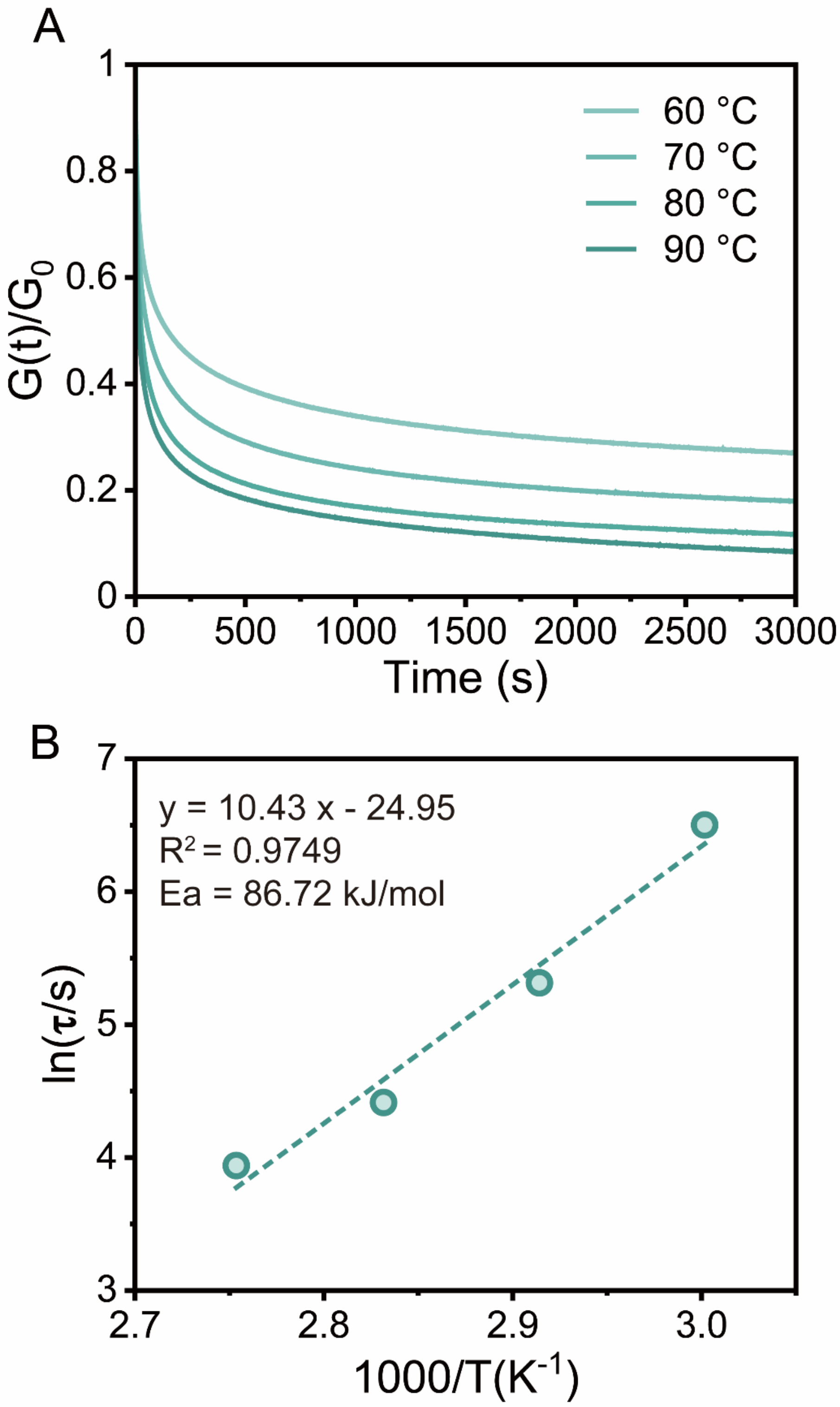

Fig. S38 **Normalized variable temperature stress relaxation of DAG-PU-2** (A) Stress relaxation curves at 60 °C, 70 °C, 80 °C and 90 °C of DAG-PU-2. (B) Plots of ln (τ*) -1000/T and linear fit and E_a for DAG-PU-2.

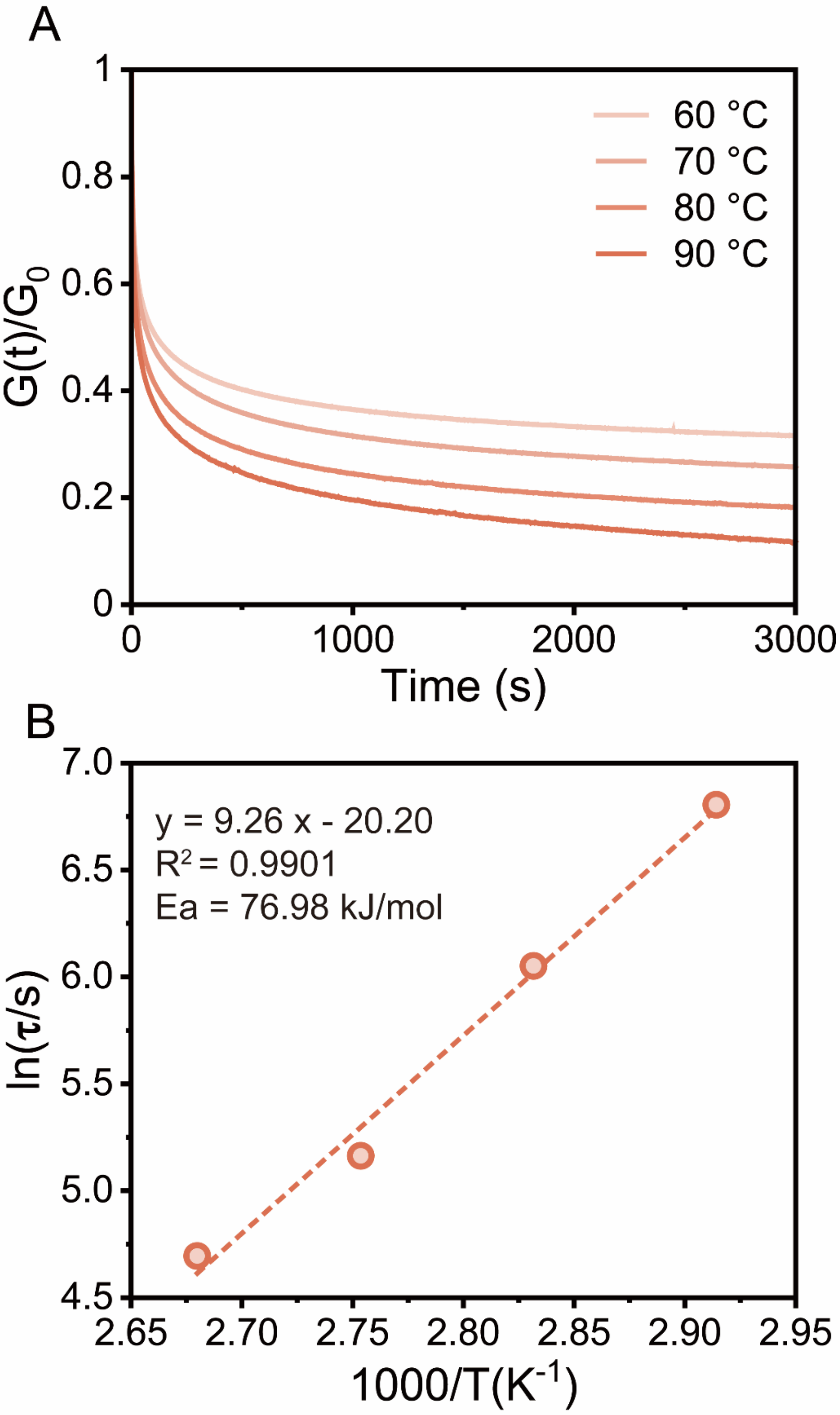

Fig. S39 **Normalized variable temperature stress relaxation of DAG-PU-3** (A) Stress relaxation curves at 60 °C, 70 °C, 80 °C and 90 °C of DAG-PU-3. (B) Plots of ln (τ*) -1000/T and linear fit and E_a for DAG-PU-3.
